# New hybrid pyridine–1,2,4-triazole scaffolds: synthesis, in vitro evaluation of anticancer and antimicrobial activity, and in silico insights

**DOI:** 10.1038/s41598-026-53201-3

**Published:** 2026-05-22

**Authors:** Aida Šermukšnytė, Dainius Lukauskas, Maryna Stasevych, Viktor Zvarych, Olena Komarovska-Porokhnyavets, Kristina Kantminienė, Ingrida Tumosienė, Vilma Petrikaitė

**Affiliations:** 1 Department of Organic Chemistry, Kaunas University of Technology, Radvilėnų pl. 19, Kaunas, 50254 Lithuania; 2 Department of Drug Targets Histopathology, Institute of Cardiology, Lithuanian University of Health Sciences, Kaunas, 50162 Lithuania; 3 Department of Technology of Biologically Active Substances, Pharmacy, and Biotechnology, Lviv Polytechnic National University, S. Bandera Str. 12, Lviv, 79013 Ukraine; 4 Department of Automated Control Systems, Lviv Polytechnic National University, S. Bandera Str. 12, Lviv, 79013 Ukraine; 5 Department of Physical and Inorganic Chemistry, Kaunas University of Technology, Radvilėnų pl. 19, Kaunas, 50254 Lithuania; 6 Institute of Biotechnology, Life Sciences Center, Vilnius University, Saulėtekio 7, Vilnius, 10257 Lithuania

**Keywords:** Acetamide, Cell viability, Cell migration, Selectivity, Tumour spheroids, Antimicrobial activity, Biochemistry, Cancer, Chemical biology, Chemistry, Computational biology and bioinformatics, Drug discovery

## Abstract

1,2,4-Triazole-3-thiones bearing 2-amino-5-chloropyridine or 2-amino-3,5-dibromopyridine moieties were *S*-alkylated with substituted bromoacetamides to obtain target 1,2,4-triazole-3-thiols. Anticancer effects of the synthesized compounds were evaluated in vitro using cell monolayer (MTT and ‘wound healing’ assays) and 3D spheroid models against human lung adenocarcinoma (A549), triple-negative breast cancer (MDA-MB-231), and melanoma (IGR39) cell lines. In the initial screening, compounds incorporating the 3,5-dibromopyridyl fragment were generally the most cytotoxic and compound **34** (5-chloropyridinyl derivative) was the most potent, with EC_50_ values below 5 µM for all tested cancer cell lines. Although cytotoxic, they did not markedly inhibit cancer cell migration. In 3D spheroid cultures, **34** significantly inhibited the growth of MDA-MB-231 and IGR39 spheroids, while compounds **24** (phenyl) and **26** (*p*-tolyl) decreased spheroid viability without altering their size. Compound **26** also demonstrated notable antimicrobial properties, exhibiting stronger antifungal activity against *C. tenuis* (7.8 µg/mL) than nystatin (15.6 µg/mL) and matching the antibacterial potency of vancomycin against *M. luteum* (7.8 µg/mL). Molecular docking suggested possible MEK/BRAF-related interactions within the mitogen-activated protein kinase pathway. Compound **34** showed the most favourable predicted binding profile for MEK (-11.134 kcal/mol) and BRAF (–10.336 kcal/mol). However, these in silico findings require to be confirmed experimentally.

**Supplementary Information:**

The online version contains supplementary material available at https://doi.org/10.1038/s41598-026-53201-3.

## Introduction

Cancer is one of the primary causes of human mortality globally, attributable to its aggressive nature and the complexities involved in early diagnosis^1^. Cancer arises from the rapid, abnormal, and uncontrollable proliferation of various cell types within the body, leading to the identification of over a hundred distinct cancer types, each exhibiting considerable variability in behaviour and treatment response^2^.

Another important global health challenge in the 21st century is antimicrobial resistance (AMR), which represents a significant and escalating global threat to public health, clinical practice, and economic stability^3^. AMR not only complicates therapeutic interventions, but also contributes to prolonged hospitalizations, increased healthcare costs, and elevated mortality rates. As traditional antibiotics become progressively ineffective, the development of novel therapeutic strategies and the discovery of new antimicrobial agents have been identified as critical solutions.

Recent advancements in synthetic chemistry and molecular design have facilitated the development of an alternative strategy to address the global health challenges mentioned above. This promising new strategy involves the creation of hybrid compounds, which integrate functional moieties capable of interacting with multiple specific biological targets. Both anticancer and antimicrobial drugs share common targets and mechanisms of action. Research has demonstrated that antibiotics can induce cancer cell apoptosis, inhibit tumour growth, and prevent cancer metastasis. Consequently, antibiotics are being increasingly utilized as adjuncts in cancer treatment.

**Fig. 1:**
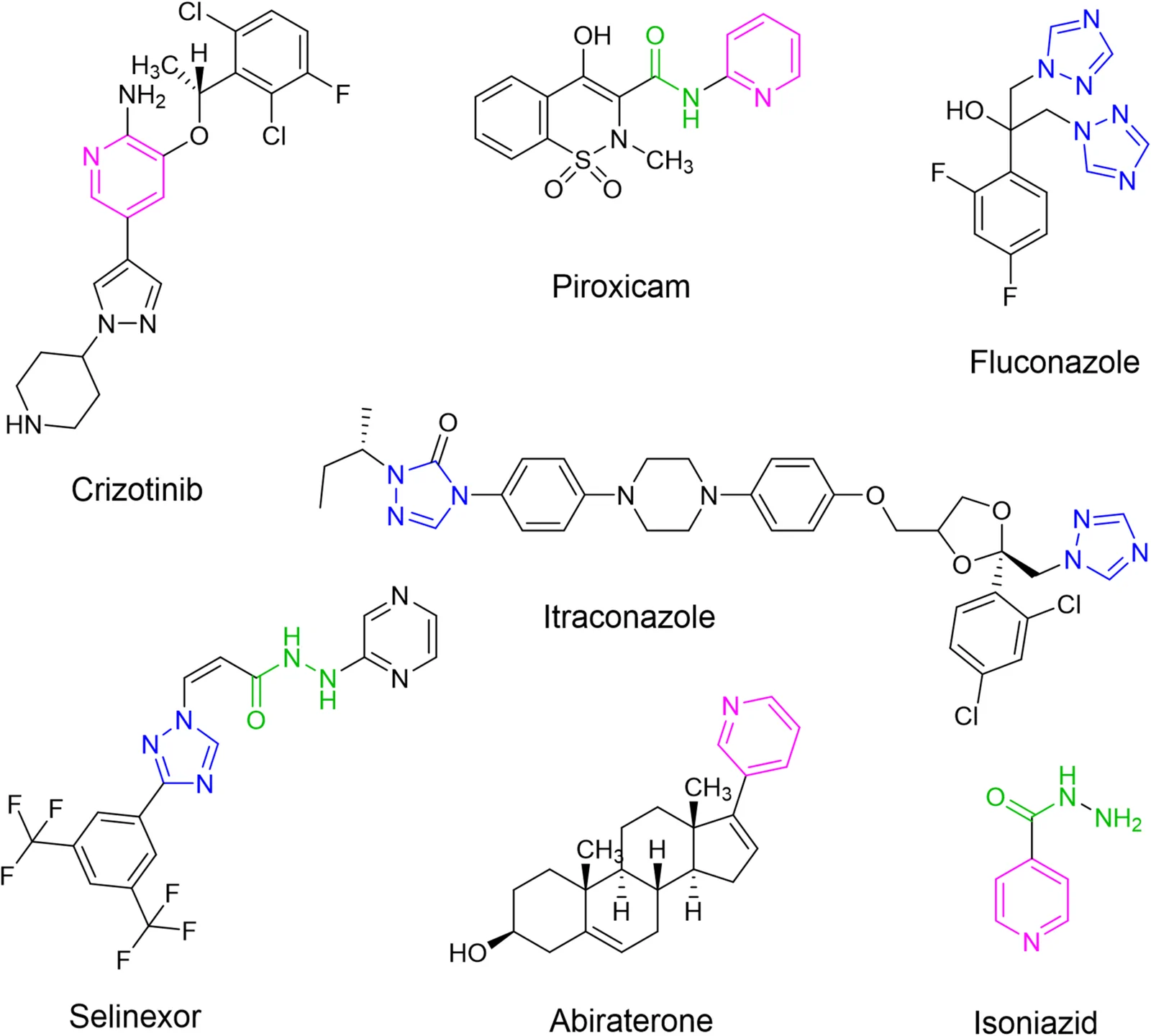
Structures of the approved drugs bearing pyridine and triazole scaffolds.

Pyridine derivatives have gained significant attention in medicinal chemistry due to their diverse biological activities, such as antiviral, antibacterial, antifungal, anticancer, and anti-inflammatory^4–6^. The US FDA database includes several approved pharmaceuticals derived from pyridine or dihydropyridine, such as abiraterone (anticancer), crizotinib (anticancer), and isoniazid (antitubercular), etc^5^. (Fig. 1). The pyridine scaffold plays a crucial role in modulating biological interactions through hydrogen bonding, π-stacking, and metal coordination. The potential of pyridine-based compounds as effective anticancer agents by targeting key enzymes, DNA interactions, and apoptotic pathways, thereby inhibiting tumour growth and proliferation has made them promising drug candidates^7,8^.

Pyridine derivatives have emerged as potent kinase inhibitors, which interact with the ATP-binding site of kinases, effectively blocking the phosphorylation process, and, thus, playing a crucial role in targeted cancer therapies and other diseases driven by aberrant kinase activity^9^. Targeting protein kinases is a widely used and highly effective strategy in cancer therapy due to their essential role in mediating cellular signal transduction and regulating key biological processes, including proliferation, survival, apoptosis, metabolism, transcription, differentiation, and various other cellular functions^10^. Several pyridine-based small-molecule kinase inhibitors, such as imatinib and crizotinib, which target multiple tyrosine kinases, have been developed and approved for treating non-small cell lung cancer (NSCLC), chronic myeloid leukaemia (CML), acute lymphocytic leukaemia (ALL), gastrointestinal stromal tumors (GIST), and other malignancies. Pyridine derivatives also exhibit strong antimicrobial properties against a wide range of bacterial and fungal pathogens, often disrupting cell wall synthesis, protein function, or nucleic acid metabolism^11^. 4-Pyridinecarbohydrazide, known as isoniazid, is an antibiotic used to treat tuberculosis. Caerulomycin and collismycin represent 2,2’-bipyridine-based natural antibiotics^12^.

1,2,4-Triazole is another significant nitrogen-containing scaffold in medicinal chemistry, widely recognized for its diverse biological activities, including antimicrobial and anticancer effects^13–17^. The 1,2,4-triazole framework serves as a key pharmacophore, exhibiting strong interactions with biological receptors due to its dipolar nature, hydrogen bonding capabilities, structural rigidity, and enhanced solubility^18^. The distinctive structure of triazole enables a range of noncovalent interactions with biological targets, allowing it to function as both a hydrogen bond donor and acceptor^19^. Triazole compounds primarily exhibit their biological effects by inhibiting enzymes such as dihydrofolate reductase, DNA gyrase, sterol 14-α-demethylase, and DNA polymerase. In addition to their enzymatic inhibition, these compounds offer several advantages, including low cytotoxicity, high bioavailability, and excellent stability and resistance to cleavage^17^. 1,2,4-Triazole derivative selinexor (Fig. 1), in combination with other pharmaceuticals, is currently approved for the treatment of multiple myeloma, a type of cancer formed from antibody-producing plasma cells. Triazole-based antifungal therapeutic compounds include fluconazole and itraconazole. Sulfur-substituted triazole derivatives, including mercapto- and thione-substituted variants, have demonstrated enhanced potency compared to their unsubstituted counterparts^20^. The 1,2,4-triazole-3-thione and 1,2,4-triazole-3-thiol structures have been associated with a broad spectrum of biological activities, including anticancer, antimicrobial, and antitubercular^19–25^.

**Fig. 2:**
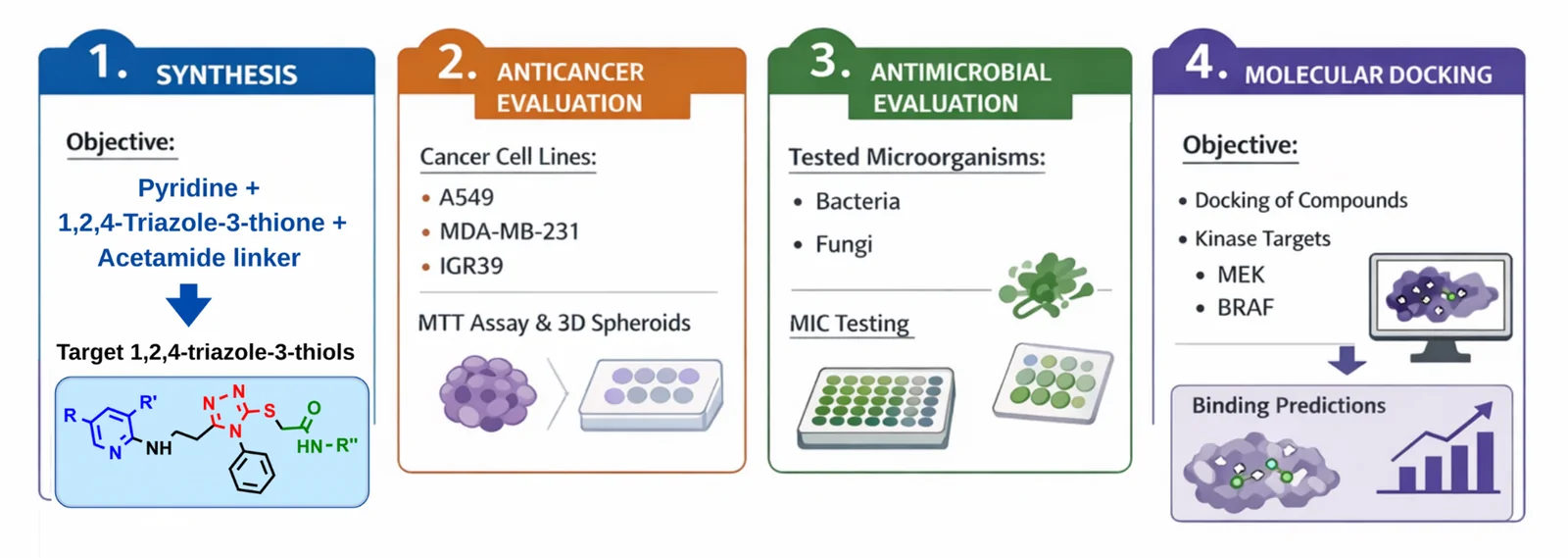
Study design and overall workflow of the research.

Based on the above information, as a continuation of our search for potent hybrid anticancer and antimicrobial drug candidates^26–28^, we herein report the synthesis of a series of acetamide derivatives bearing pyridine and 1,2,4-triazole-3-thiol moieties and in vitro evaluation of their anticancer and antimicrobial activity supported by in silico assessment (Fig. 2).

For testing anticancer activity, we selected three different cancer cell lines, representing human non-small cell lung adenocarcinoma (A549), triple-negative breast cancer (MDA-MB-231), and malignant melanoma (IGR39). These cell lines were deliberately selected to evaluate whether the compounds used in the study could be potentially useful as therapeutic agents against tumours of different origins, characterised by drug resistance. Treatment options for triple-negative breast cancer are limited, as these tumours lack both hormonal and HER2 receptors^29^; therefore, further investigation and the search for potential targeted therapeutic agents are required. Lung cancer is often detected and diagnosed only at advanced stages^30^, when treatment options become limited. For this reason, it is crucial to further develop both diagnostic approaches and chemotherapy for lung cancer, as surgical treatment is frequently not feasible after diagnosis and/or is associated with poor survival outcomes^31^. Non-small cell lung cancer accounts for the majority of all cases (80–85%)^30^. Melanoma accounts for only 1% of all skin cancer cases; however, it is characterized by a highly aggressive course and is responsible for approximately 80% of deaths among all skin cancer types^32,33^. Despite therapeutic advances in melanoma treatment, a proportion of tumours do not respond to available therapies due to intrinsic or acquired resistance^34^. Together with the challenges of ensuring accurate early diagnosis^35^, this highlights the need to explore alternative treatment strategies and novel therapeutic agents. We selected these three cell lines for our study as they represent aggressive, metastasis-prone, and often therapy-resistant cancer types. Each of them is characterized by specific mutations that contribute to drug resistance and increased proliferation.

## Results and discussion

### Chemistry

**Scheme 1:**
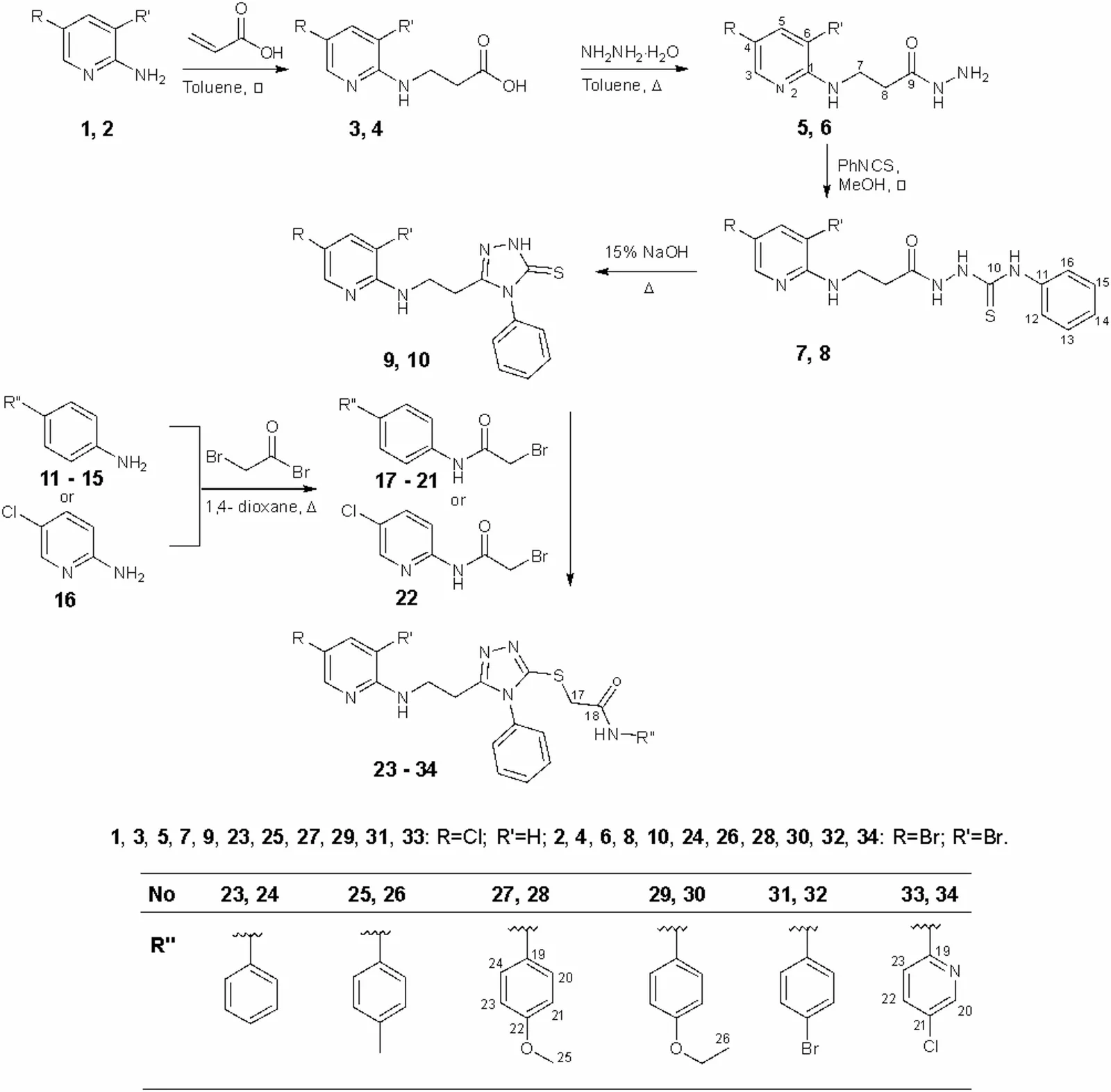
Synthesis of novel 1,2,4-triazole-3-thiols **23–34**.

The target 1,2,4-triazole-3-thiols **23–34** were synthesized via a pathway shown in Scheme 1. Reaction of aminopyridines **1** and **2** with acrylic acid in toluene under reflux conditions provided respective carboxylic acids **3** and **4**, which underwent reaction with hydrazine monohydrate in toluene to afford hydrazides **5** and **6**. Semicarbazides **7** and **8** were synthesized from the respective hydrazides **5** and **6** through the reaction with phenyl isothiocyanate in methanol under reflux conditions, followed by alkaline cyclization to afford 1,2,4-triazole-3-thiones **9** and **10**, respectively. In the ^1^H NMR spectra of **7** and **8**, proton of the NH group in aminopyridine moiety resonated at 6.77 ppm and 6.55 ppm, respectively, and protons of NH groups in semicarbazide moiety gave three singlets at approx. 9.5 ppm and 10 ppm. In the ^13^C NMR spectra of **9** and **10**, the carbon resonances attributed to the 1,2,4-triazole-3-thione C = S carbon (167.6 ppm and 167.4 ppm, respectively) are shifted downfield in comparison to the signals of the corresponding carbon atoms in semicarbazides **7** and **8** (173.6 ppm and 173.5 ppm, respectively). The final reaction step constituted the attachment of the synthesized bromoacetamides **17–22** to 1,2,4-triazole-3-thiones **9** and **10**, providing the target *S*-alkylated products **23–34**. ^1^H NMR spectrum of **33**, displays the double set of singlets at 9.54 ppm and 10.95 ppm attributed to the NH proton in the intensity ratio 0.3:0.7. Although compounds **23–34** are structurally related, the splitting of the NH signal into two singlets is observed only for **33**. This behavior can be attributed to a unique combination of structural and electronic factors that enable the formation of two slowly interconverting amide rotamers in the DMSO-*d*_*6*_ solution. The presence of a double signals for the NH proton in the ^1^H NMR spectrum of **33** suggests the formation of the favourable intramolecular hydrogen bond between the amide NH and the pyridine nitrogen, stabilizing one conformer and increasing the energy barrier for rotation around the C–N bond^36^. In other synthesized target compounds, slight differences in substituents likely prevent the formation of such a stabilized conformation or allow faster interconversion, resulting in a single averaged NH signal. The bromoacetamides **17–21**, containing unsubstituted and differently substituted phenyl ring in their structure, were synthesized by the reaction of amines **11–15** and bromoacetyl bromide in 1,4-dioxane in the presence of triethylamine at room temperature as described in^37^. Bromoacetamide **22** bearing a pyridine moiety was synthesized via an analogous three-step synthesis procedure from the starting compound 2-amino-5-chloropyridine (**16**).

In the ^13^C NMR spectra of **23–34**, the carbon attached to S atom (C-S) in the 1,2,4-triazole ring resonated at 154.5–154.8 ppm, i.e. at a lower field than the respective carbons in 1,2,4-triazole-3-thione C = S group in **9** and **10**.

### Biology

#### Compound effect on cell viability

**Fig. 3:**
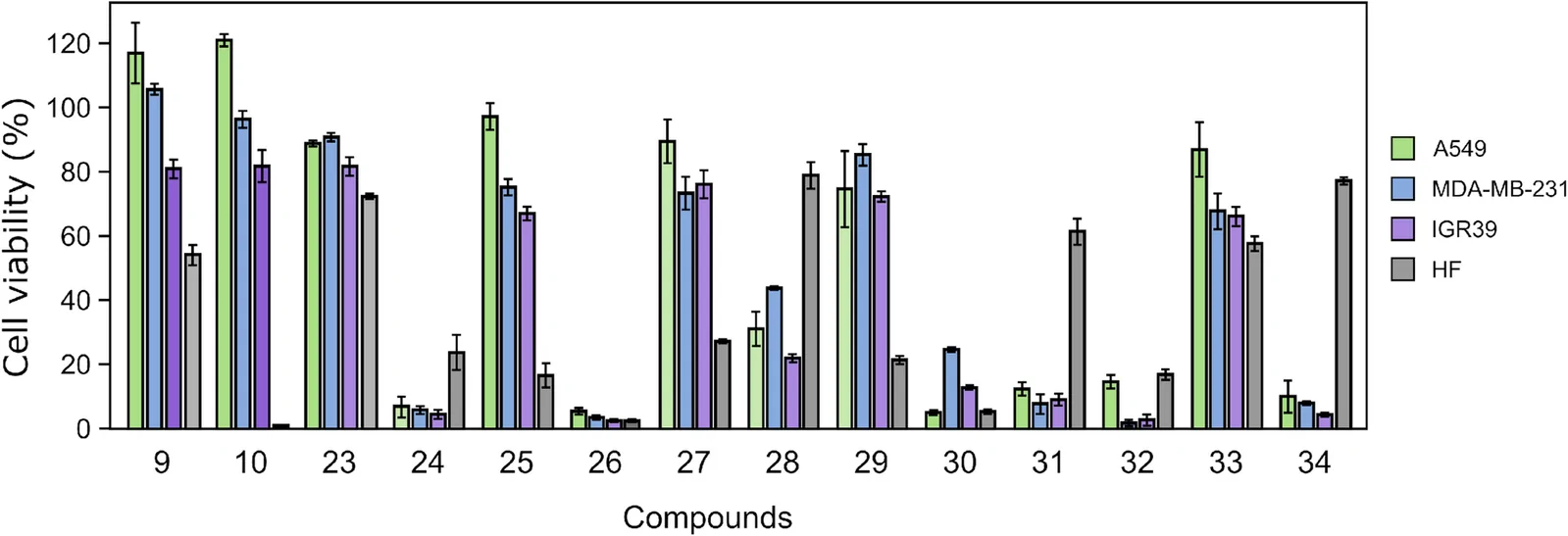
Effect of compounds **9**, **10**, and **23**–**34** at a 50 µM concentration on the viability of human lung adenocarcinoma A549 cells, human triple-negative breast cancer MDA-MB-231 cells, human melanoma IGR39 cells and human fibroblasts, HFs, *n* = 3. MTT assay results after 72 h of incubation.

At a 50 µM concentration, most compounds demonstrated comparable effects across the various cancer cell lines. Six compounds – **24**, **26**, **30**, **31**, **32**, and **34** – were identified as the most active, reducing cancer cell viability to below 20% (Fig. 3). Among these, *N*-(4-bromophenyl)-2-((5-(2-((5-chloropyridin-2-yl)amino)ethyl)-4-phenyl-4*H*-1,2,4-triazol-3-yl)thio)acetamide (**31**) and *N*-(5-chloropyridin-2-yl)-2-((5-(2-((3,5-dibromopyridin-2-yl)amino)ethyl)-4-phenyl-4*H*-1,2,4-triazol-3-yl)thio)acetamide (**34**) showed the most favourable selective effects. Specifically, 5-chloropyridyl derivative with 4-bromophenyl fragment **31** resulted in viability ranging from 5 to 11% in MDA-MB-231 cells and 58 to 66% in HF, while 3,5-dibromopyridyl derivative bearing the 5-chloropyridyl fragment **34** reduced the viability of cells to 4.68 ± 0.26% viability in IGR39 cells and 77.75 ± 1.16% in HF. 3,5-Dibromopyridyl derivative with the 4-ethoxyphenyl fragment **30** exhibited the strongest cytotoxic activity in the A549 cell line, but the weakest in MDA-MB-231. Compounds **26** and **30** were retained for further study despite displaying comparable cytotoxicity in fibroblasts and cancer cells, owing to their significant overall reduction in cancer cell viability. However, the lack of selectivity observed for these at 50 µM represents a notable limitation. At this concentration, compounds such as **26** and **30** reduced fibroblast viability to below 20%, comparable to their effects on cancer cells, yielding selectivity indices (SI = EC₅₀ in HF / EC₅₀ in cancer cells) near or below 1 for most compound-cell line combinations, indicating that the compounds were comparably toxic to fibroblasts and cancer cells at the tested concentrations. However, several factors inherent to the 2D monolayer format may cause an underestimation of selectivity. In standard 2D culture, fibroblasts proliferate actively, whereas in vivo they exist predominantly in a quiescent state within connective tissues^38^. Since most cytotoxic agents preferentially kill dividing cells, 2D assays may overestimate fibroblast sensitivity. Moreover, Howes et al. demonstrated that the selective cytotoxicity of anticancer agents against cancer cells over normal fibroblasts was observed specifically in 3D co-culture spheroids but not in 2D co-culture monolayers of the same cell populations^39^. This underscores that 2D-derived selectivity indices may not accurately predict the in vivo therapeutic window and that complementary 3D models are essential for a more informative assessment. A general trend indicated that most 1,2,4-triazole-3-thiols reduced the viability of IGR39 cells the most and featured a lower inhibitory effect on A549 and MDA-MB-231 cells.

The structure-activity relationship (SAR) analysis revealed that the anticancer activity was notably influenced by the halogenation patterns on both the pyridine fragments and the arylacetamide substituents. Five of the six compounds selected for further studies (**24**, **26**, **30**, **32**, and **34**) bear the 3,5-dibromopyridyl fragment, while **31** contains the 5-chloropyridyl fragment. A general trend indicated that dibrominated analogues were typically more cytotoxic than their chlorinated counterparts. For instance, 3,5-dibromopyridyl derivative **26** was significantly more active than its structural analogue **25** (5-chloropyridyl derivative with the *p*-tolyl fragment). However, a clear activity trend between structural analogues with the same arylacetamide substituent but differing in 5-chloropyridyl and 3,5-dibromopyridyl fragments was not consistently observed, except for **31** and **32**. Both **31** and **32** had a bromo substituent in the arylacetamide fragment and showed similar activity against cancer cells, but **32** (with a 3,5-dibromopyridyl fragment) exhibited lower selectivity, causing approximately a threefold greater viability reduction in human fibroblasts than **31**. This suggests that while one brominated aromatic fragment is necessary for significant anticancer activity, additional brominated aromatic fragments or their position may reduce selectivity for cancer cells.

**Fig. 4:**
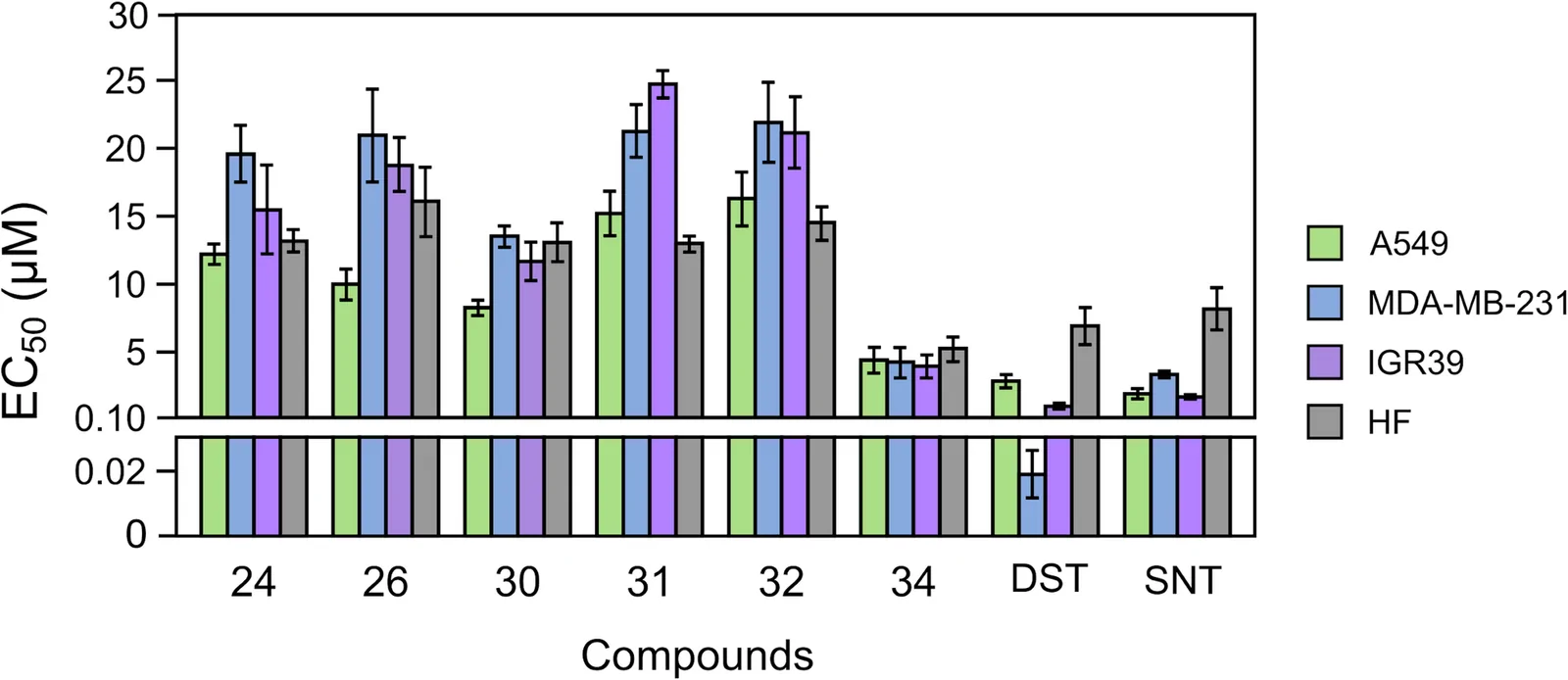
The EC_50_ values of selected compounds, dasatinib (DST) and sunitinib (SNT) in human lung adenocarcinoma A549, human triple-negative breast cancer MDA-MB-231, human melanoma IGR39 and human fibroblasts, HF, *n* = 3. MTT assay results after 72 h of incubation.

Following the initial screening, the half-maximal effective concentration (EC_50_) values were determined for the most active compounds and compared to those of the clinically approved reference drugs, dasatinib and sunitinib (Fig. 4; Table 1). All novel triazole derivatives generally exhibited higher EC_50_ values, indicating lower potency compared to both sunitinib and dasatinib. The novel compounds featured the least cytotoxicity consistently against MDA-MB-231 cells, while the strongest effects were noted in A549 cells.

**Table 1:** EC₅₀ values (µM) of selected compounds, dasatinib (DST), and sunitinib (SNT), determined by the MTT assay after 72 h of incubation. Data are presented as mean ± SD, *n* = 3.

Compound	A549	MDA-MB-231	IGR39	HF
**24**	12.20 ± 0.69	19.57 ± 2.10	15.47 ± 3.23	13.17 ± 0.76
**26**	10.00 ± 1.14	20.97 ± 3.40	18.73 ± 1.94	16.10 ± 2.52
**30**	8.27 ± 0.50	13.53 ± 0.75	11.67 ± 1.40	13.07 ± 1.38
**31**	15.20 ± 1.61	21.23 ± 1.96	24.73 ± 1.03	13.00 ± 0.56
**32**	16.30 ± 1.97	21.90 ± 2.98	21.13 ± 2.69	14.53 ± 1.21
**34**	4.40 ± 0.89	4.27 ± 1.11	3.97 ± 0.83	5.27 ± 0.87
DST	2.87 ± 0.40	0.019 ± 0.007	1.01 ± 0.13	6.93 ± 1.33
SNT	1.93 ± 0.35	3.36 ± 0.11	1.70 ± 0.20	8.17 ± 1.50

EC₅₀, half-maximal effective concentration; A549, human lung adenocarcinoma; MDA-MB-231, human triple-negative breast cancer; IGR39, human melanoma; HF, human fibroblasts; DST, dasatinib; SNT, sunitinib.

Compound **34** emerged as the most active among the newly synthesized compounds with EC_50_ values for cancer cell lines below 5 µM. Molecular docking studies suggest that optimal activity of **34** is linked to its high affinity for MEK kinase, with its docking score to MEK surpassing that of trametinib, a known MEK inhibitor (Table 4). However, when compared to dasatinib, EC_50_ values of **34** were approximately 2–4 times higher in IGR39 and A549 cells, and, notably, 200 times higher in MDA-MB-231 cells. A similar trend is seen when compared to sunitinib, with it being approximately 4 times more active across all cancer cell lines. Although the novel compounds lack potency compared to sunitinib and dasatinib, therapeutic agents possessing higher EC_50_ values are actively used in practice, such as dacarbazine, with EC_50_ values ranging from 25 to 100 µM in melanoma cell lines^40,41^.

Compounds **31** and **32** were ultimately excluded from further evaluation due to their preferential toxicity towards fibroblasts. All triazole compounds did not display a particular selectivity towards cancer cells in 2D models, however, that is not to say it translates to equivalent in vivo activity. Considering the difficulties in assessing selectivity in 2D models, as discussed in a previous publication^26^, promising candidates cannot be determined solely by EC_50_ values. Therefore, we decided to continue conducting experiments with the selected compounds using cell migration and spheroid activity assays, which would better reflect the in vivo tumour environment^42^.

### Compound effect on cell migration

**Fig. 5:**
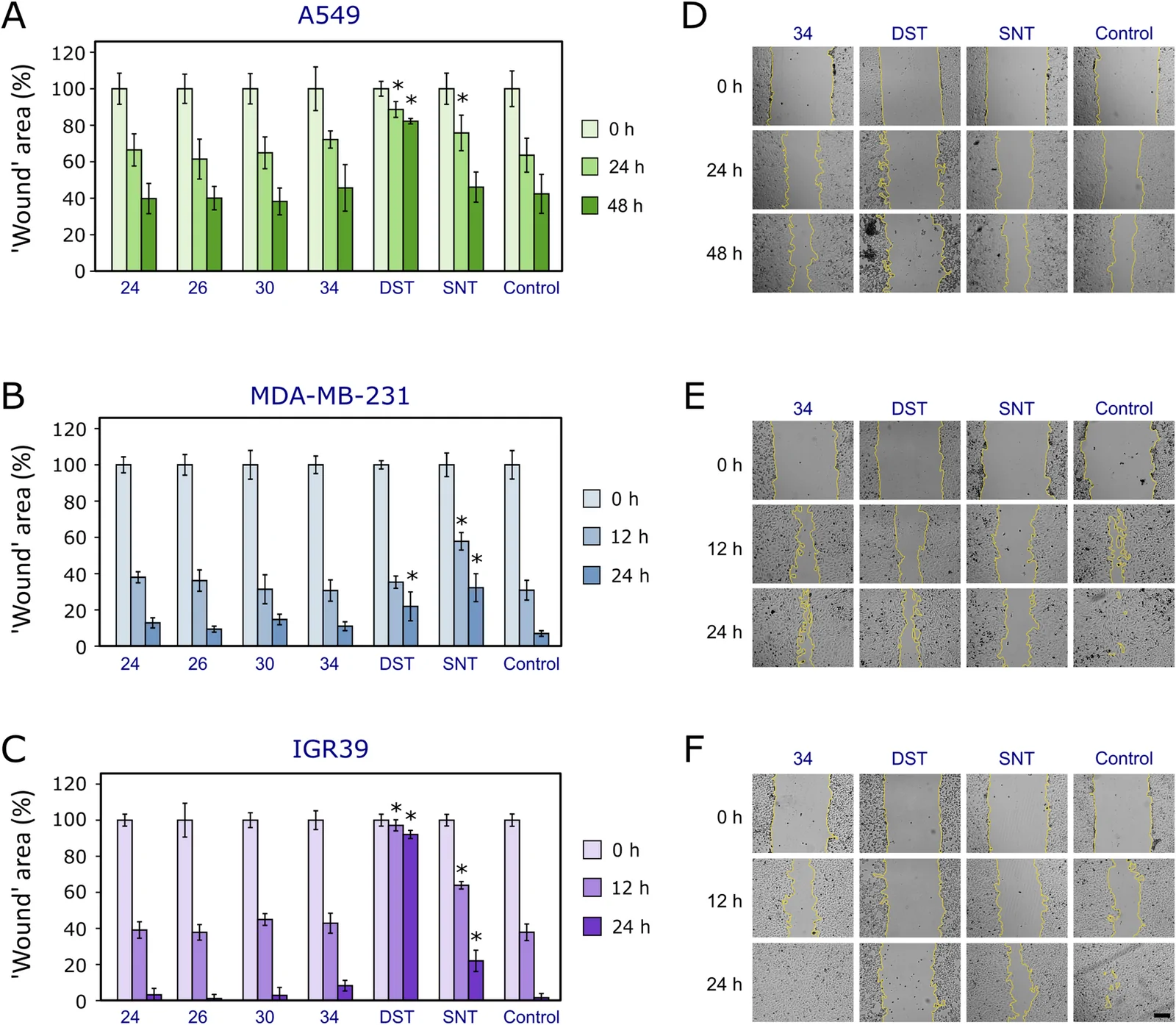
Effects of compounds **24**, **26**, **30**, **34**, dasatinib (DST), and sunitinib (SNT) on human lung adenocarcinoma A549 (**A**), human triple-negative breast cancer MDA-MB-231 (**B**), and human melanoma IGR39 (**C**) migration at 50% of their respective EC_50_ concentrations, *n* = 3. Photographs of the ‘wound’ areas in A549, MDA-MB-231, and IGR39 monolayers treated with compounds at 50% of respective EC_50_ values at the indicated time points of the experiment (**D**). Edges of the ‘wound’ are highlighted with a yellow line. Scale bar indicates 200 μm. Statistical significance is denoted with an asterisk (*) for values *p* < 0.05.

The effect of the selected 1,2,4-triazole-3-thiol derivatives (**24**, **26**, **30**, and **34**) on cancer cell migration was evaluated at concentrations equivalent to 50% and 10% of their respective EC_50_ values, chosen to either minimize general cytotoxicity (10% EC_50_) or assess effects distinct from or synergistic with general cytotoxicity (50% EC_50_) (Fig. 5).

Only the reference compounds (sunitinib and dasatinib) produced a statistically significant effect on A549, MDA-MB-231 and IGR39 cell lines at their respective partial EC_50_ concentrations. Dasatinib, a known Src/FAK inhibitor^43^, almost entirely halted the migration of IGR39 and A549 cells (‘wound’ area was above 80% at all time points). Both sunitinib and dasatinib inhibited migration the least in MDA-MB-231 cells. It is worth noting that dasatinib in similar experiments displayed more significant results in MDA-MB-231 cells; however, concentrations five times higher than ours were used^43,44^.

The general failure of the novel 1,2,4-triazole-3-thiol derivatives to inhibit cell migration at both 10% (Figure S57) and 50% (Fig. 5) of their respective EC_50_ values points to a functional dissociation in their anticancer activity. This can be seen in an opposite example, including betulin-triazole derivatives, with their IC_50_ values averaging to approximately 35 µM, but achieving significant migration reduction in ‘wound healing’ assays at 10 µM concentrations^45^. This suggests a different mechanism of action, potentially one that does not target pathways critical to cell migration or is incompatible with the migration mechanisms of the selected cell lines, or an interaction akin to vemurafenib’s (BRAF^V600^ inhibitor) paradoxical MAPK pathway activation^46,47^. Molecular docking results indicated that the novel 1,2,4-triazole-3-thiols exhibit lower affinity for FAK/Src kinases, which are known to be crucial for migration in MDA-MB-231 cells^48,49^, thus anticipating a limited effect on motility. The primary impact of the novel 1,2,4-triazole-3-thiol derivatives is likely associated with the inhibition of cell proliferation, rather than directly impeding cell migration. Furthermore, variations in structural substituents or the level of halogenation did not yield any clear trends in this study regarding their impact on cell migration.

### Compound effect in 3D cell cultures

Spheroids were generated using the magnetic 3D bioprinting method, co-culturing cancer cells with human fibroblasts in a 1:1 ratio to better represent the tumour microenvironment^50^.

**Fig. 6:**
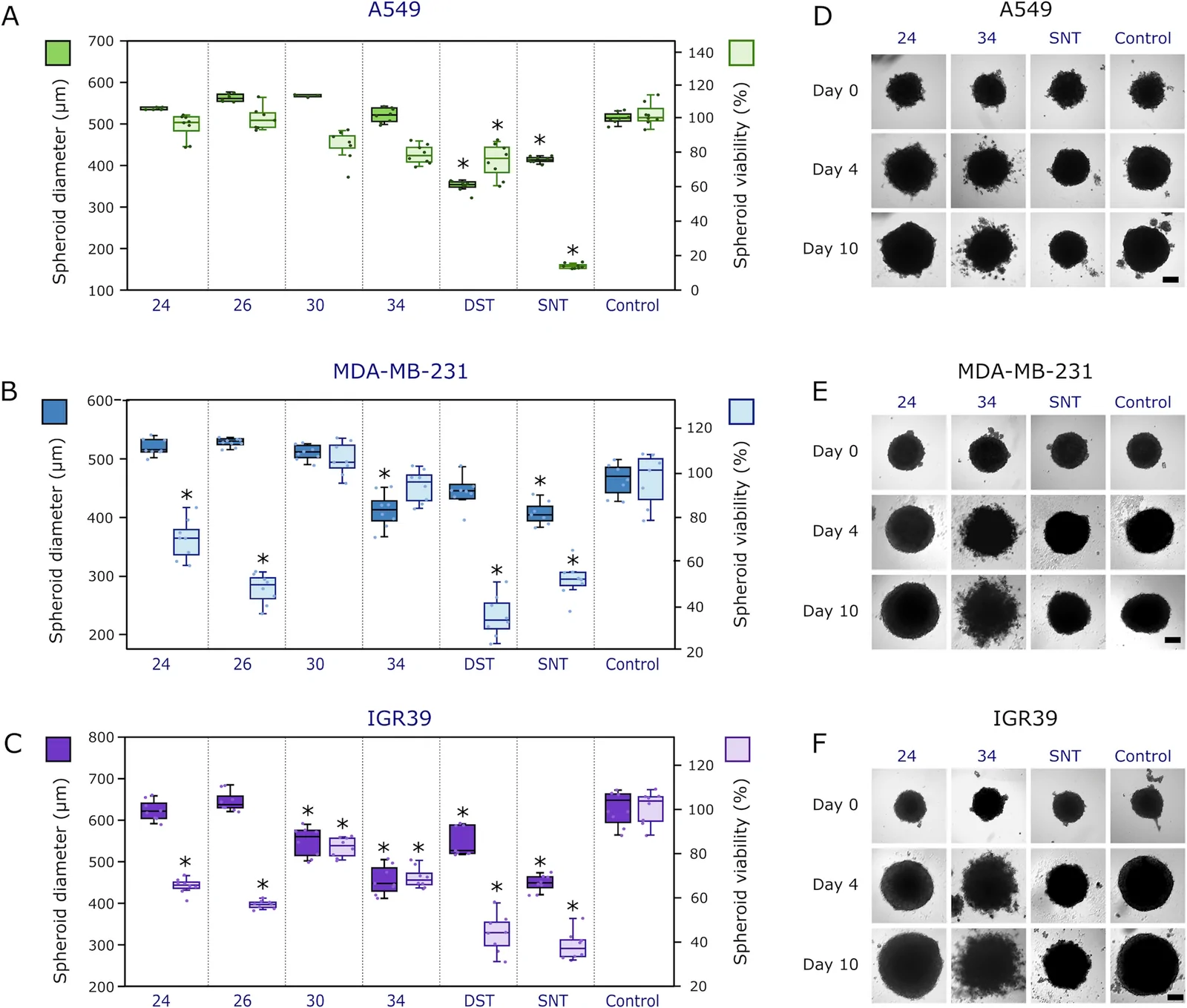
Effect of compounds **24**, **26**, **30**, **34**, dasatinib (DST), and sunitinib (SNT) on human lung adenocarcinoma A549 (**A**), human triple negative breast cancer MDA-MB-231 (**B**), human melanoma IGR39 (**C**) spheroid viability (indicated by a brighter color) and size (indicated by a darker color). Representative images of spheroids at days 0, 4 and 10 are provided to the right of the graphs (**D**–A549, **E**–MDA-MB-231, **F**–IGR39). Statistical significance: (*) denotes *p* < 0.05 versus untreated controls. Inner dashes indicate medians, and whiskers illustrate maximum and minimum values. A scatterplot of all individual data points is overlaid over each bar. Scale bar = 200 μm.

A fixed compound concentration of 10 µM was applied for all treatments, chosen based on the average EC_50_ values of the 1,2,4-triazole-3-thiol derivatives and the tendency of kinase inhibitors to accumulate in tissues in vivo at concentrations significantly higher than their plasma levels^51,52^(Fig. 6). We can hypothesize that the formed spheroids contained a hypoxic core, considering the starting size of spheroids^53^(spheroid diameter at day 0 ranged from 300 to 400 μm across all cell lines (Fig. 6D, E, F).

In general, both the 1,2,4-triazole-3-thiol derivatives and the reference compounds exhibited lower activity in 3D cultures compared to 2D monolayers, a common observation attributed to the complex tumour microenvironment within spheroids seen in both our own^26^ and other publications^54^. For A549 spheroids, the novel compounds did not significantly inhibit either spheroid growth or viability. This is partly expected, as the A549 cell line harbours a KRAS mutation, which leads to a constitutive activation of the MAPK pathway independent of BRAF^55–57^; however, inhibiting targets further downstream, such as targeting MEK, as is the case with trametinib, does produce significant results at nanomolar concentrations^58^. Despite compound **34** possessing a higher affinity for the MEK kinase active site than trametinib (Table 4), similar potency in practice is not observed. In contrast, sunitinib significantly decreased A549 spheroid size (− 27.5 ± 1.7% decrease) and viability (9.99 ± 1.56%). Dasatinib also significantly inhibited both spheroid viability and growth, albeit to a lesser extent than sunitinib.

In MDA-MB-231 spheroids, **34** significantly inhibited spheroid growth, resulting in a spheroid diameter approximately 7.4% smaller compared to the control. However, this growth inhibition occurred with minimal impact on overall cell viability. This differential effect, where growth is halted but cells remain largely viable, is reminiscent of the cytostatic action of MEK inhibitors, which can arrest tumour cells in the G_1_ phase^59^. Conversely, compounds **26** and **24** significantly reduced cell viability in MDA-MB-231 spheroids (to approximately 55–56% viability) without significantly altering the overall spheroid size. This suggests that **26** and **24**, with their more neutral aromatic substituents (*p*-tolyl and phenyl), might induce direct cell death without immediate structural collapse. This can be observed in other studies involving cytotoxic agents, such as docetaxel^60^; however, further studies are needed for clarification.

In IGR39 spheroids, **34** exhibited significant growth inhibition, reducing spheroid diameter by approximately 30%, which was more pronounced than the effect of dasatinib. However, **34** reduced spheroid viability to a lesser extent (approximately 77.8% viable cells) compared to its effects on growth. Like MDA-MB-231 spheroids, **26** and **24** significantly reduced IGR39 spheroid viability (to approximately 55.6%) but did not significantly affect spheroid size. Consistent with the novel triazole compounds’ affinity for BRAF mutants (Table 4), every tested compound significantly inhibited the viability of IGR39 spheroids, albeit to a lower extent than a standalone BRAF^V600^ and MEK inhibitor combination, such as vemurafenib (BRAF inhibitor) and cobimetinib (MEK inhibitor) in melanoma cell lines^61^.

A notable morphological change observed was the early cell detachment from the edges of A549, MDA-MB-231, and IGR39 spheroids treated with **34**, beginning as early as day 4 of the experiment. This effect was not observed with any other 1,2,4-triazole-3-thiol derivative in the series (Figures S58-S60). As such, spheroid growth assessment for spheroids treated with compound **34** is more complex and may be less accurate. Such morphological changes may point to potential effects on cell adhesion or intercellular interactions. This effect is likely attributable to the 2-pyridyl acetamide group in the structure of **34**, as other dibrominated compounds **24**–**32** did not cause similar detachment.

### Antimicrobial activity

The antimicrobial activity of the synthesized compounds was evaluated against Gram-negative and Gram-positive strains of the bacteria *E. coli*,* S. aureus*, and *M. luteum*, as well as the fungal strains *C. tenuis* and *A. niger*. In the first step, the agar diffusion method was used to evaluate the antimicrobial properties at 0.5% and 0.1% concentrations. Vancomycin (antibacterial drug) and nystatin (antifungal drug) were used as reference drugs (control).

As seen from the results in Table 2, which represent the diameters of the growth inhibition zones of microbial strains, among the synthesized compounds, six compounds exhibited favourable outcomes. It should be noted that the only active 2-amino-5-chloropyridine derivative was compound 1,2,4-triazole-3-thione **9**. None of the 1,2,4-triazole-3-thiols bearing a 2-amino-5-chloropyridinyl moiety was active against the tested bacterial and fungal strains. 1,2,4-triazole-3-thiols, bearing 2-amino-3,5-dibromopyridinyl and 4-methylphenyl moiety **26**, phenyl ring **24**, and 4-bromophenyl moiety **32** exhibited high antibacterial effect against bacterium *M. luteum* in comparison with that of vancomycin at 0.5% concentration (the diameter of inhibition zones of microbial growth d = 22–25 mm). Compounds **24** and **26** at 0.5% concentration exhibited greater activity against *E. coli* than the reference drug (the diameter of the inhibition zones of microbial growth d = 16–17 mm). 1,2,4-triazole-3-thiol **24** acted as a better growth inhibitor of *S. aureus* than vancomycin. The antifungal activity test has revealed 1,2,4-triazole-3-thiols, bearing 4-methylphenyl **26** and 4-bromophenyl **32** rings, to exhibit high antifungal activity against fungus strain *C. tenuis* at 0.5% concentration (d = 22–25 mm), which is higher than that of the reference drug nystatin. The antifungal activity of compounds **26**, **24**, and **32** was lower than that of the reference drug. 1,2,4-Triazole-3-thiones **9** and **10** as well as compound **28** did not suppress the growth of tested fungal strains at concentrations of 0.5 and 0.1% (d = 0 mm).

**Table 2:** Antimicrobial effect of the synthesized compounds determined by the diffusion technique.

Compound	Concentration, %	Diameter of inhibition zones of microorganism growth d, mm				
		E. coli	S. aureus	M. luteum	C. tenuis	A. niger
**9**	0.5	0	0	12.0	0	0
	0.1	0	0	0	0	0
**10**	0.5	0	0	10.0	0	0
	0.1	0	0	0	0	0
**24**	0.5	17.0	17.0	22.0	15.0	12.0
	0.1	0	0	12.0	0	0
**26**	0.5	16.0	0	25.0	26.0	14.0
	0.1	0	0	10.0	14.0	0
**28**	0.5	0	0	8.0	0	0
	0.1	0	0	0	0	0
**32**	0.5	0	13.0	25.0	28.0	12.0
	0.1	0	0	15.0	13.0	0
Vancomycin	0.5	14.0	15.0	18.0	–	–
Nystatin	0.5	–	–	–	19.0	20.0

Only compounds with positive results are presented in the table.

In the second step of the evaluation of antimicrobial properties of the synthesized compounds, the minimum inhibitory concentrations (MICs) were determined by the serial dilution method with concentrations ranging from 0.9 to 500.0 µg/mL. As it could be expected, the inhibitory effect of 1,2,4-triazole-3-thiols **26** and **32** on the test culture *M. luteum* is at the same level as the control (for **26**) or close to it, with MIC values at 7.8 µg/mL and 15.6 µg/mL, respectively (Table 3). It is worth noting that the growth of this bacterial strain was also suppressed by other active compounds at MIC values ranging from 31.2 µg/mL to 125.0 µg/mL. Compounds **26** and **32** possess higher antifungal activity against *C. tenuis* (MIC = 7.8 µg/mL) than that of nystatin, while 24 inhibited the growth of this fungal strain at the same MIC as the control (MIC = 15.6 µg/mL). The effect of these compounds on the *A. niger* strain was found to be within the range of MIC = 7.8–31.2 µg/mL, which is lower than that of the control.

**Table 3:** Minimal inhibitory concentrations (MICs) of the active synthesised compounds determined by the serial dilution technique.

Compound/test culture	MIC, µg/mL				
	E. coli	S. aureus	M. luteum	C. tenuis	A. niger
**9**	500.0	500.0	62.5	+	+
**10**	+	+	125.0	+	+
**24**	62.5	62.5	31.2	15.6	15.6
**26**	+	+	7.8	7.8	31.2
**28**	+	+	31.2	+	+
**32**	125.0	31.2	15.6	7.8	7.8
Vancomycin**	3.9	7.8	7.8	−	−
Nystatin	−	−	−	15.6	3.9

Only compounds with positive results are presented in the table.

^+^growth of the microorganism was observed in the studied concentrations.

In summary, the evaluation of the antimicrobial activity of the synthesized compounds has revealed that 1,2,4-triazole-3-thiols **24**, **26**, and **32** are promising derivatives that exhibited a high antibacterial effect against the bacteria *M. luteum* and antifungal activity against the fungus *C. tenuis*.

### Molecular docking study

The next stage of our study included molecular docking for compounds **24**, **26**, **30**, **31**, **32**, and **34** and the known multikinase inhibitors sunitinib and dasatinib (reference drugs), which demonstrated significant experimental results against the *MDA-MB-231*, IGR39 and *A549* cancer cell lines. The study was conducted to identify a plausible anticancer mechanism.

An initial docking screen against canonical sunitinib targets was performed for compounds **24**, **26**, **30**, **31**, **32**, and **34** (Table S1, Supporting Information). The results showed that these compounds exhibited consistently less favourable predicted binding than sunitinib itself. Their docking scores ranged from − 4.487 to − 7.659 kcal/mol, whereas sunitinib showed markedly stronger predicted binding, with values from − 10.163 to − 11.892 kcal/mol. As this analysis did not support a sunitinib-like target profile for the studied compounds, these targets were not considered further in the main discussion. We therefore focused the subsequent analysis on protein kinases for which the tested compounds showed more favourable interactions, particularly BRAF and MEK.

Next stage of docking study for the compounds **24**, **26**, **30**, **31**, **32**, and **34** was carried out to the structures of target proteins EGFR (PDB: 4HJO), VEGFR (PDB: 4AGD, 4ASD, 3EWH), HER2 (PDB: 3RCD), BRAF (PDB: 1UWH, 5VAM, 4G9C), MEK (PDB: 4U7Z), SCR (PDB: 3F3V), CDK5 (PDB: 1UNL). The studied compounds have the best affinity to the next protein targets: BRAF (PDB: 1UWH, 5VAM, 4G9C) and MEK (PDB: 4U7Z). The structures of kinase drugs Vemurafenib (BRAF-inhibitor), Trametinib (MEK-inhibitor) for melanoma IGR39, and dasatinib (BRAF-inhibitor, reference drug) for breast MDA-MB-231 cancer and Sorafenib (BRAF-inhibitor) lung cancer A549 were used as being binding to the respective kinases. As can be seen from the docking results (Table 4), Sunitinib has a low affinity to BRAF/MEK serine threonine protein kinases (docking score − 5.607…-8.287 kcal/mol). It should be noted that the literature currently has no data on experimental studies of sunitinib as a BRAF or MEK inhibitor.

**Fig. 7:**
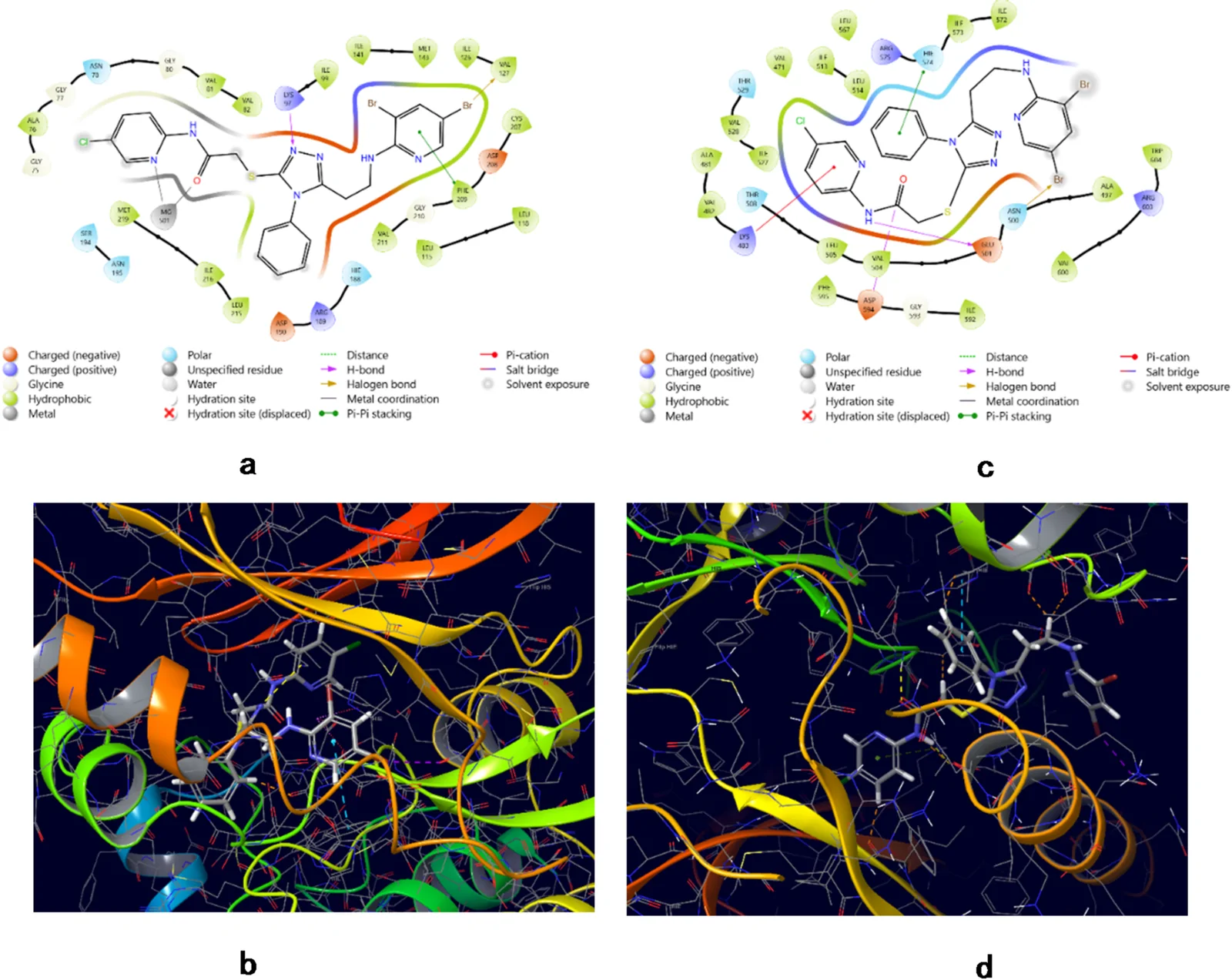
The 2D and 3D interactions of compound **34** with amino acid residues in the active sites of proteins: (**a**,** b**)— with MEK (4U7Z); (**c**,** d**)— with BRAF (PDB: 4G9C).

Results showed that two compounds had a high level of binding in the next serine threonine protein kinases sites: compound **32** to *5VAM (BRAF)*, and compound to **34** *1UWH*,* 4G9C* (BRAF), and *4U7Z* (MEK). The compound **34** had the highest affinity for 4U7Z (docking score − 11.134 kcal/mol, glide emodel − 162.081 kcal/mol) in comparison to Trametinib and other kinase drugs (Table 4). The binding of compound **34** at the protein site 4U7Z is presented as the 2D and 3D visualisations in Fig. 7a, b. Also, **34** showed docking score and glide emodel parameters to *5VAM and 4G9C* (*BRAF*, Fig. 7c, d) on the level of Sorafenib.

**Table 4:** Docking score values of **24**, **26**, **30**, **31**, **32**, and **34** ligands and reference ligands interaction with serine threonine protein kinases.

Ligand	Serine threonine protein kinases							
	BRAF (PDB: 1UWH)		BRAF (PDB: 5VAM)		BRAF (PDB: 4G9C)		MEK (PDB: 4U7Z)	
	Docking Score kcal/mol	Glide Emodel kcal/mol	Docking Score kcal/mol	Glide Emodel kcal/mol	Docking Score kcal/mol	Glide Emodel kcal/mol	Docking Score kcal/mol	Glide Emodel kcal/mol
**24**	− 8.078	− 88.095	− 8.756	− 88.274	− 9.800	− 111.386	− 9.926	− 135.960
**26**	− 8.070	− 91.821	− 8.523	− 86.552	− 9.765	− 97.087	− 8.372	− 110.012
**30**	− 8.236	− 86.385	− 9.825	− 95.602	− 10.299	− 89.495	− 9.279	− 129.434
**31**	− 8.543	− 87.750	− 9.947	− 102.685	− 9.810	− 105.315	− 9.624	− 124.952
**32**	− 8.951	− 87.717	− **10.259**	− **104.741**	− 9.935	− 93.003	− 8.820	− 125.059
**34**	− **9.805**	− **92.582**	− 8.710	− 95.712	− **10.336**	− **113.386**	− **11.134**	− **162.081**
Sunitinib	− 7.152	− 68.226	− 8.287	− 56.178	− 7.216	− 60.730	− 5.607	− 56.774
Dasatinib	− **10.428**	− **101.336**	− **8.593**	− **87.010**	− **9.956**	− **94.452**	− 8.052	− 104.318
Sorafenib	− **10.093**	− **122.715**	− **10.349**	− **98.181**	− **10.676**	− **109.879**	− 6.406	− 88.934
Trametinib	− 10.256	− 62.904	− 5.498	− 29.961	− 5.873	− 37.082	− **9.545**	− **111.156**
Vemurafenib	− **11.313**	− **96.824**	− **10.285**	− **94.962**	− **10.136**	− **90.640**	− 7.275	− 89.902

Significant values are in bold.

Therefore, the molecular docking results suggest that compound **34** may interact with MEK- and BRAF-related targets within the mitogen-activated protein kinase pathway. However, these in silico observations require direct experimental validation in kinase-based assays before any definitive mechanistic conclusions can be drawn. This may provide a key direction for further research focused on finding new and potent anticancer drugs for treating breast, lung cancer, and melanoma.

## Methods

### Chemistry

#### General conditions

Reagents were purchased from Sigma-Aldrich (St. Louis, MO, USA) and TCI Europe N.V. (Zwijndrecht, Belgium). The reaction course and purity of the synthesized compounds were monitored by TLC using aluminium plates precoated with silica gel 60 F254 (MerckKGaA, Darmstadt, Germany). The ^1^H and ^13^C NMR spectra were recorded in DMSO-*d*_*6*_ on a Bruker Avance III (400 MHz, 101 MHz) spectrometer (Bruker BioSpin AG, Fällanden, Switzerland) operating in the Fourier transform mode. Chemical shifts (*δ*) are reported in parts per million (ppm) calibrated from TMS (0 ppm) as an internal standard for ^1^H NMR, and DMSO-*d*_*6*_ (39.43 ppm) for ^13^C NMR. FT-IR spectra (ν, cm^− 1^) were recorded on a Perkin–Elmer Spectrum BX FT–IR spectrometer (Perkin–Elmer Inc., Waltham, MA, USA) using KBr pellets. Mass spectra were obtained on a Bruker maXis UHR-TOF mass spectrometer (Bruker Daltonics, Bremen, Germany) with positive ESI ionization. The melting points were determined on a MEL-TEMP (Electrothermal, A Bibby Scientific Company, Burlington, NJ, USA) melting point apparatus and are uncorrected.

#### Synthesis

*3-((5-Chloropyridin-2-yl)amino)propanoic acid (* ***3*** *)* was synthesized as described in^62^. The ^1^H and ^13^C NMR spectra were found to be identical to the ones described in^62^.

##### 3-((3,5-Dibromopyridin-2-yl)amino)propanoic acid (**4**)

To a solution of **2** (18 mmol) in toluene (20 mL), acrylic acid (18 mmol) was added and the reaction mixture was heated under reflux for 10 h. After cooling, the excess toluene was decanted. Then, aqueous 10% NaOH solution (20 mL) and ethanol (20 mL) were added to the reaction mixture, and heating under reflux was continued for 2 h. The hot mixture was filtered off, and the filtrate was allowed to cool to room temperature. It was acidified with HCl until pH 5–6. The precipitate was recrystallized from ethanol, filtered, and dried. Yield 4.27 g (83%), brown crystals, m.p. 126–127 °C. ^1^H NMR (400 MHz, DMSO-*d*_*6*_): δ = 2.47 (s, 2 H, H_8_), 3.41 (s, 2 H, H_7_), 6.78 (s, 1H, NH), 7.65–7.71 (m, 2 H, H_3,5_), 14.59 (s, 1H, H_9_).^13^C NMR (101 MHz, DMSO-*d*_*6*_): δ = 35.4 (C_8_), 37.9 (C_7_), 105.1, 110.6, 139.0, 147.8, 157.7, 174.9 (C_1,3,4,5,6,9_).

##### 3-((5-Chloropyridin-2-yl)amino)propanehydrazide (**5**)

To **3** (50 mmol) dissolved in toluene (75 mL), hydrazine monohydrate (50 mmol) was added, and the reaction mixture was heated under reflux for 4 h using a Dean-Stark trap for azeotropic separation of formed water. After the reaction was complete, the excess toluene was decanted, and the residue was dissolved in propan-2-ol (100 mL). The precipitate was filtered off and recrystallized from propan-2-ol. Yield 4.27 g (40%), white crystals, m.p. 95–96 °C. ^1^H NMR (400 MHz, DMSO-*d*_*6*_): δ = 2.28 (t, 2 H, *J* = 6.8 Hz, H_8_), 3.41 (q, 2 H, *J* = 6.8 Hz, *J* = 13.2 Hz, H_7_), 3.75 (s, 2 H, NH_2_), 6.48 (d, 1H, *J* = 8.8 Hz, H_6_), 6.70 (t, 1H, *J* = 6.0 Hz, NH), 7.39 (d, 1H, *J* = 9.2 Hz, H_5_), 7.93 (s, 1H, H_3_), 9.03 (s, 1H, NH). ^13^C NMR (101 MHz, DMSO-*d*_*6*_): δ = 33.6 (C_8_), 37.7 (C_7_), 109.9, 117.6, 136.6, 145.6, 157.4, 170.0 (C_1,3,4,5,6,9_).

##### 3-((3,5-Dibromopyridin-2-yl)amino)propanehydrazide (**6**)

To **4** (15 mmol) dissolved in toluene (50 mL), hydrazine monohydrate (15 mmol) was added, and the reaction mixture was heated under reflux for 4 h using a Dean-Stark apparatus for azeotropic separation of formed water. After the reaction was complete, the excess toluene was decanted, and the resulting precipitate was filtered off and recrystallized from propan-2-ol to yield the purified product. Yield 3.43 g (66%), white crystals, m.p. 204–205 °C. ^1^H NMR (400 MHz, DMSO-*d*_*6*_): δ = 2.33 (t, 2 H, *J* = 7.2 Hz, H_8_), 3.51 (q, 2 H, *J* = 6.4 Hz, *J* = 12.8 Hz, H_7_), 5.76 (s, 1H, NH), 6.58–6.72 (m, 2 H, NH_2_), 7.99 (s, 1H, H_5_), 8.11 (s, 1H, H_3_), 9.04 (s, 1H, NH). ^13^C NMR (101 MHz, DMSO-*d*_*6*_): δ = 32.7 (C_8_), 38.0 (C_7_), 104.4, 105.1, 141.0, 146.8, 153.5, 170.0 (C_1,3,4,5,6,9_).

##### 2-(3-((5-Chloropyridin-2-yl)amino)propanoyl)-N-phenylhydrazine-1-carbothioamide (**7**)

To **5** (9.3 mmol) dissolved in methanol (30 mL), phenyl isothiocyanate (10 mmol) was added, and the reaction mixture was stirred under reflux for 7 h. Afterwards, the mixture was cooled, the precipitate was filtered off and recrystallized from ethanol and water mixture. Yield 1.73 g (55%), white crystals, m.p. 144–145 °C. IR (KBr) ν_max_ (cm^− 1^): 1512 (C = S), 1695 (C = O), 3332–3166 (4 NH). ^1^H NMR (400 MHz, DMSO-*d*_*6*_): δ = 3.50 (s, 4 H, H_7,8_), 6.52 (d, 1H, *J* = 8.8 Hz, H_6_), 6.77 (t, 1H, *J* = 5.8 Hz, NH), 7.18 (t, 1H, *J* = 7.2 Hz, H_14_), 7.35 (t, 2 H, *J* = 8.8 Hz, H_13_,_15_), 7.42–7.45 (m, 3 H, H_5_,_12_,_16_), 7.98 (s, 1H, H_3_), 9.53 (s, 2 H, 2NH), 9.97 (s, 1H, NH). ^13^C NMR (101 MHz, DMSO-*d*_*6*_): δ = 33.5 (C_8_), 37.3 (C_7_), 110.0, 117.7, 128.4, 136.8, 139.2, 145.6, 157.4 (C_1,3,4,5,6,9, 11−16_), 173.6 (C_10_). MS (ESI^+^): *m/z* calculated for C_15_H_16_ClN_5_OS, 350 [M + H]^+^; found 350.

##### 2-(3-((3,5-Dibromopyridin-2-yl)amino)propanoyl)-N-phenylhydrazine-1-carbothioamide (**8**)

To **6** (6 mmol) dissolved in methanol (30 mL), phenyl isothiocyanate (7.5 mmol) was added. The reaction mixture was heated under reflux for 1 h. Then, it was cooled, the precipitate was filtered off and recrystallized from ethanol and water mixture. Yield 2.05 g (74%), white crystals, m.p. 163–164 °C. IR (KBr) ν_max_ (cm^− 1^): 1493 (C = S), 1676 (C = O), 3392–2921 (4 NH). ^1^H NMR (400 MHz, DMSO-*d*_*6*_): δ = 3.70 (s, 4 H, H_7,8_), 6.55 (t, 1H, *J* = 6.2 Hz, NH), 7.16 (t, 1H, *J* = 7.4 Hz, H_14_), 7.32 (t, 2 H, *J* = 7.6 Hz, H_13,15_), 7.39 (d, 2 H, *J* = 7.6 Hz, H_12,16_), 7.97 (s, 1H, H_3_), 8.10 (s, 1H, H_5_), 9.55 (s, 2 H, 2NH), 9.94 (s, 1H, NH). ^13^C NMR (101 MHz, DMSO-*d*_*6*_): δ = 33.0 (C_8_), 37.6 (C_7_), 104.8, 105.4, 128.4, 139.3, 141.4, 147.0, 153.7 (C_1,3,4,5,6,9, 11−16_), 173.5 (C_10_). MS (ESI^+^): *m/z* calculated for C_15_H_15_Br_2_N_5_OS, 474 [M + H]^+^; found 474.

##### 5-(2-((5-Chloropyridin-2-yl)amino)methyl)-4-phenyl-2,4-dihydro-3 H-1,2,4-triazole-3-thione (**9**)

Compound **7** (3 mmol) was dissolved in 30 mL of 15% aqueous NaOH, and the reaction mixture was stirred under reflux for 4 h. After completion, the solution was cooled to room temperature and subsequently acidified with HCl until crystallization occurred. The precipitate was recrystallized from DMF/H_2_O mixture. Yield 0.88 g (93%), brown crystals, m.p. 119–120 °C. IR (KBr) ν_max_ (cm^− 1^): 1419 (C = S), 3240, 3286 (2 NH). ^1^H NMR (400 MHz, DMSO-*d*_*6*_): δ = 2.65 (t, 2 H, *J* = 6.8 Hz, H_8_), 3.35 (q, 2 H, *J* = 6.8 Hz, *J* = 13.2 Hz, H_7_), 6.39 (d, 1H, *J* = 8.8 Hz, H_6_), 6.90 (t, 1H, *J* = 6.0 Hz, NH), 7.35–7.37 (m, 3 H, H_5,13,15_), 7.47–7.54 (m, 3 H, H_12,14,16_), 7.82 (s, 1H, H_3_), 13.73 (s, 1H, NH_triazole_). ^13^C NMR (101 MHz, DMSO-*d*_*6*_): δ = 25.7 (C_8_), 37.9 (C_7_), 110.1, 117.6, 128.5, 129.6, 133.8, 136.6, 145.4, 150.9, 157.1 (C_1,3,4,5,6,9,11−16_), 167.6 (C_10_). MS (ESI^+^): *m/z* calculated for C_15_H_14_ClN_5_S, 332 [M + H]^+^; found 332. Anal. Calcd. (%) for C_15_H_14_ClN_5_S: C, 54.30; H, 4.25; N, 21.11; S, 9.66. Found: C, 54.01; H, 4.36; N, 21.39; S, 9.54.

##### 5-(2-((3,5-Dibromopyridin-2-yl)amino)methyl)-4-phenyl-2,4-dihydro-3 H-1,2,4-triazole-3-thione (**10**)

Mixture of **8** (1 g, 2 mmol) and 15% aqueous NaOH (20 mL) was heated under reflux for 4 h. After the reaction was complete, the reaction mixture was cooled down to room temperature and acidified with HCl, leading to the formation of a solid precipitate. The precipitate was recrystallized from DMF/H_2_O mixture. Yield 0.93 g (94%), white crystals, m.p. 279–280 °C. IR (KBr) ν_max_ (cm^− 1^): 1504 (C = S), 2891, 3348 (2 NH). ^1^H NMR (400 MHz, DMSO-*d*_*6*_): δ = 2.66 (t, 2 H, *J* = 6.8 Hz, H_8_), 3.45 (q, 2 H, *J =* 6.8 Hz, *J* = 12.8 Hz, H_7_), 6.65 (t, 1H, *J* = 5.8 Hz, NH), 7.30 (d, 2 H, *J* = 7.2 Hz, H_12,16_), 7.44–7.47 (m, 3 H, H_13,14,15_), 7.89 (s, 2 H, H_3,5_). ^13^C NMR (101 MHz, DMSO-*d*_*6*_): δ = 25.5 (C_8_), 39.0 (C_7_), 104.8, 105.4, 128.6, 129.5, 129.7, 134.4, 141.5, 146.8, 151.0, 153.7 (C_1,3,4,5,6,9,11−16_), 167.4 (C_10_). MS (ESI^−^): *m/z* calculated for C_15_H_13_Br_2_N_5_S, 454 [M-H]^+^; found 454. Anal. Calcd. (%) for C_15_H_13_Br_2_N_5_S, C, 39.58; H, 2.88; N, 15.39; S, 7.04. Found: C, 39.69; H, 2.72; N, 15.35; S, 6.99.

2-Bromo-*N*-phenylacetamides **17**–**22** were prepared as described in^37^. The ^1^H and ^13^C NMR spectra were found to be identical to the ones described in^37^.

##### General procedure for the synthesis of compounds **23–34**

To a solution of 1,2,4-triazolethione **9** or **10** (9.0 mmol or 7.0 mmol, respectively) in acetone (30 mL), the corresponding amide (9.0 mmol or 7.0 mmol, respectively) and K₂CO₃ (9.0 mmol or 7.0 mmol, respectively) were added, and the reaction mixture was stirred at 40 °C for 5 h. After the reaction was complete, distilled water was added until the precipitate formed. The precipitate was recrystallized from propan-2-ol.

##### 2-((5-(2-((5-Chloropyridin-2-yl)amino)ethyl)-4-phenyl-4 H-1,2,4-triazol-3-yl)thio)-N-phenylacetamide (**23**)

Prepared from 2-bromo-*N*-phenylacetamide. Yield 0.27 g (64%), light pink crystals, m.p. 178–179 °C. IR (KBr) ν_max_ (cm^− 1^): 1596 (C = O), 3074, 3267 (2 NH). ^1^H NMR (400 MHz, DMSO-*d*_*6*_): δ = 2.77 (t, 2 H, *J* = 6.8 Hz, H_8_), 3.45 (q, 2 H, *J* = 6.8 Hz, *J* = 13.2 Hz, H_7_), 4.08 (s, 2 H, H_17_), 6.38 (d, 1H, *J* = 8.8 Hz, H_6_), 6.83 (t, 1H, *J* = 6.0 Hz, NH), 7.05 (t, 1H, *J* = 7.2 Hz, H_22_), 7.28–7.35 (m, 3 H, H_5,20,24_), 7.38–7.41 (m, 2 H, H_12,16_), 7.52–7.55 (m, 5 H, H_13,14,15,21,23_), 7.82 (s, 1H, H_3_), 10.37 (s, 1H, NH). ^13^C NMR (101 MHz, DMSO-*d*_*6*_): δ = 24.9 (C_8_), 37.1 (C_7_), 38.8 (C_17_), 110.0, 117.6, 119.3, 123.8, 127.5, 129.0, 130.1, 133.0, 136.6, 138.9, 145.4, 149.8, 154.4, 157.0, 165.8 (C_1,3,4,5,6,9−16,18−24_). MS (ESI^+^): *m/z* calculated for C_23_H_21_ClN_6_OS, 465 [M + H]^+^; found 465. Anal. Calcd. (%) for C_23_H_21_ClN_6_OS, C, 59.41; H, 4.55; N, 18.07; S, 6.90. Found: C, 59.49; H, 4.62; N, 18.10; S, 6.97.

##### 2-((5-(2-((3,5-Dibromopyridin-2-yl)amino)ethyl)-4-phenyl-4 H-1,2,4-triazol-3-yl)thio)-N-phenylacetamide (**24**)

Prepared from 2-bromo-*N*-phenylacetamide. Yield 0.25 g (66%), white crystals, m.p. 160–161 °C. IR (KBr) ν_max_ (cm^− 1^): 1591 (C = O), 2979, 3386 (2 NH). ^1^H NMR (400 MHz, DMSO-*d*_*6*_): δ = 2.80 (t, 2 H, *J* = 7.0 Hz, H_8_), 3.53 (d, 2 H, *J* = 6.4 Hz, H_7_), 4.04 (s, 2 H, H_17_), 6.64 (s, 1H, H_22_), 7.03–7.07 (m, 1H, NH), 7.27–7.37 (m, 5 H, H_5,20,21,23,24_), 7.50–7.55 (m, 5 H, H_12−16_), 7.88 (s 1H, H_3_), 10.32–10.42 (m, 1H, NH). ^13^C NMR (101 MHz, DMSO-*d*_*6*_): δ = 24.7 (C_8_), 30.6 (C_7_), 37.2 (C_17_), 104.9, 105.5, 119.6, 124.2, 127.6, 129.3, 130.3, 133.0, 139.0, 141.5, 146.9, 150.1, 153.6, 165.5, 166.1 (C_1,3,4,5,6,9−16,18−24_). MS (ESI^+^): *m/z* calculated for C_23_H_20_Br_2_N_6_OS, 589 [M + H]^+^; found 589. Anal. Calcd. (%) for C_23_H_20_Br_2_N_6_OS, C, 46.96; H, 3.43; N, 14.29; S, 5.45. Found: C, 46.75; H, 3.39; N, 14.25; S, 5.57.

##### 2-((5-(2-((5-Chloropyridin-2-yl)amino)ethyl)-4-phenyl-4 H-1,2,4-triazol-3-yl)thio)-N-(p-tolyl)acetamide (**25**)

Prepared from 2-bromo-*N*-(*p*-tolyl)acetamide. Yield 0.25 g (58%), brownish crystals, m.p. 189–190 °C. IR (KBr) ν_max_ (cm^− 1^): 1596 (C = O), 3068, 3271 (2 NH). ^1^H NMR (400 MHz, DMSO-*d*_*6*_): δ = 2.23 (s, 3 H, H_25_), 2.77 (t, 2 H, *J* = 7.0 Hz, H_8_), 3.45 (d, 2 H, *J* = 6.8 Hz, H_7_), 4.06 (s, 2 H, H_17_), 6.37 (d, 1H, *J* = 8.8 Hz, H_6_), 6.82 (t, 1H, *J* = 6.0 Hz, NH), 7.10 (d, 2 H, *J* = 8.4 Hz, H_20,24_), 7.34–7.54 (m, 8 H, H_5,12−16,21,23_), 7.81 (s, 1H, H_3_), 10.25 (s, 1H, NH). ^13^C NMR (101 MHz, DMSO-*d*_*6*_): δ = 20.6 (C_25_), 24.9 (C_8_), 37.0 (C_7_), 38.8 (C_17_), 110.0, 117.7, 119.3, 127.5, 129.4, 130.1, 132.8, 133.0, 136.6, 145.4, 149.9, 154.4, 157.0, 165.5 (C_1,3,4,5,6,9−16,18−24_). MS (ESI^+^): *m/z* calculated for C_24_H_23_ClN_6_OS, 479 [M + H]^+^; found 479. Anal. Calcd. (%) for C_24_H_23_ClN_6_OS, C, 60.18; H, 4.84; N, 17.55; S, 6.69. Found: C, 60.25; H, 4.85; N, 17.58; S, 6.74.

##### 2-((5-(2-((3,5-Dibromopyridin-2-yl)amino)ethyl)-4-phenyl-4 H-1,2,4-triazol-3-yl)thio)-N-(p-tolyl)acetamide (**26**)

Prepared from 2-bromo-*N*-(*p*-tolyl)acetamide. Yield 0.27 g (69%), light pink crystals, m.p. 101–102 °C. IR (KBr) ν_max_ (cm^− 1^): 1585 (C = O), 3049, 3257 (2 NH). ^1^H NMR (400 MHz, DMSO-*d*_*6*_): δ = 2.22 (s, 3 H, H_25_), 2.80 (t, 2 H, *J* = 6.6 Hz, H_8_), 3.53 (q, 2 H, *J* = 6.4 Hz, *J* = 12.8 Hz, H_7_), 4.02 (s, 2 H, H_17_), 6.64 (t, 1H, *J* = 6.2 Hz, NH), 7.10 (m, 3 H, *J* = 8.4 Hz, H_13,14,15_), 7.34–7.43 (m, 5 H, H_5,20,21,23,24_), 7.51 (d, 2 H, *J* = 5.2 Hz, H_12,16_), 7.87 (s, 1H, H_3_), 10.23–10.33 (m, 1H, NH). ^13^C NMR (101 MHz, DMSO-*d*_*6*_): δ = 20.8 (C_25_), 24.7 (C_8_), 30.6 (C_7_), 37.2 (C_17_), 104.9, 105.5, 119.7, 127.6, 129.6, 130.3, 133.0, 133.6, 136.5, 141.5, 146.9, 150.2, 153.6, 154.8, 165.2, 165.8 (C_1,3,4,5,6,9−16,18−24_). MS (ESI^+^): *m/z* calculated for C_24_H_22_Br_2_N_6_OS, 603 [M + H]^+^; found 603. Anal. Calcd. (%) for C_24_H_22_Br_2_N_6_OS, C, 47.86; H, 3.68; N, 13.95; S, 5.32. Found: C, 47.92; H, 3.65; N, 13.99; S, 5.38.

##### 2-((5-(2-((5-Chloropyridin-2-yl)amino)ethyl)-4-phenyl-4 H-1,2,4-triazol-3-yl)thio)-N-(4-methoxyphenyl)acetamide (**27**)

Prepared from 2-bromo-*N*-(4-methoxyphenyl)acetamide. Yield 0.28 g (64%), white crystals, m.p. 171–172 °C. IR (KBr) ν_max_ (cm^− 1^): 1596 (C = O), 2950, 3271 (2 NH). ^1^H NMR (400 MHz, DMSO-*d*_*6*_): δ = 2.77 (t, 2 H, *J* = 6.8 Hz, H_8_), 3.45 (q, 2 H, *J* = 6.8 Hz, *J* = 13.2 Hz, H_7_), 3.70 (s, 3 H, H_25_), 4.05 (s, 2 H, H_17_), 6.37 (d, 1H, *J* = 8.8 Hz, H_6_), 6.82 (t, 1H, *J* = 6.0 Hz, NH), 6.88 (d, 2 H, *J* = 8.8 Hz, H_20,24_), 7.33–7.40 (m, 3 H, H_5,12,16_), 7.45 (d, 2 H, *J* = 8.8 Hz, H_21,23_), 7.52–7.54 (m, 3 H, H_13,14,15_), 7.82 (s, 1H, H_3_), 10.20 (s, 1H, NH). ^13^C NMR (101 MHz, DMSO-*d*_*6*_): δ = 24.9, 25.6 (C_8_), 37.0 (C_7_), 38.8 (C_17_), 55.4 (C_25_), 110.0, 114.1, 117.7, 120.9, 127.5, 130.1, 132.0, 133.0, 136.6, 145.4, 167.4 (C_1,3,4,5,6,9−16,18−24_). MS (ESI^+^): *m/z* calculated for C_24_H_23_ClN_6_O_2_S, 517 [M + Na]^+^; found 517. Anal. Calcd. (%) for C_24_H_23_ClN_6_O_2_S, C, 58.24; H, 4.68; N, 16.98; S, 6.48. Found: C, 58.39; H, 4.63; N, 17.00; S, 6.51.

##### 2-((5-(2-((3,5-Dibromopyridin-2-yl)amino)ethyl)-4-phenyl-4 H-1,2,4-triazol-3-yl)thio)-N-(4-methoxyphenyl)acetamide (**28**)

Prepared from 2-bromo-*N*-(4-methoxyphenyl)acetamide. Yield 0.29 g (71%), light brown crystals, m.p. 161–162 °C. IR (KBr) ν_max_ (cm^− 1^): 1583 (C = O), 3249, 3353 (2 NH). ^1^H NMR (400 MHz, DMSO-*d*_*6*_): δ = 2.79 (t, 2 H, *J* = 7.2 Hz, H_8_), 3.53 (d, 2 H, *J* = 7.2 Hz, H_7_), 4.00 (s, 3 H, H_25_), 4.17–4.43 (m, 2 H, H_17_), 6.64 (s, 1H, NH), 6.86 (d, 2 H, *J* = 8.8 Hz, H_20,24_), 7.35–7.36 (m, 2 H, H_13,15_), 7.42 (d, 2 H, *J* = 8.8 Hz, H_21,23_), 7.49–7.51 (m, 3 H, H_12,14,16_), 7.87 (s, 2 H, H_3,5_), 10.18 (s, 1H, NH). ^13^C NMR (101 MHz, DMSO-*d*_*6*_): δ = 24.7 (C_8_), 37.1 (C_17_), 40.1 (C_7_), 55.6 (C_25_), 104.9, 105.5, 114.3, 121.3, 127.6, 130.3, 132.1, 133.0, 141.4, 146.9, 150.1, 153.6, 154.8, 155.9, 165.6 (C_1,3,4,5,6,9−16,18−24_). MS (ESI^+^): *m/z* calculated for C_24_H_22_Br_2_N_6_O_2_S, 619 [M + H]^+^; found 619. Anal. Calcd. (%) for C_24_H_22_Br_2_N_6_O_2_S, C, 46.62; H, 3.59; N, 13.59; S, 5.18. Found: C, 46.57; H, 3.50; N, 13.51; S, 5.13.

##### 2-((5-(2-((5-Chloropyridin-2-yl)amino)ethyl)-4-phenyl-4 H-1,2,4-triazol-3-yl)thio)-N-(4-ethoxyphenyl)acetamide (**29**)

Prepared from 2-bromo-*N*-(4-ethoxyphenyl)acetamide. Yield 0.35 g (76%), brown crystals, m.p. 178–179 °C. IR (KBr) ν_max_ (cm^− 1^): 1602 (C = O), 3064, 3249 (2 NH). ^1^H NMR (400 MHz, DMSO-*d*_*6*_): δ = 1.28 (t, 3 H, *J* = 7.0 Hz, H_26_), 2.77 (t, 2 H, *J* = 7.0 Hz, H_8_), 3.44 (q, 2 H, *J* = 6.8 Hz, *J* = 13.2 Hz, H_7_), 3.95 (q, 2 H, *J* = 6.8 Hz, *J* = 14.0 Hz, H_25_), 4.04 (s, 2 H, H_17_), 6.38 (d, 1H, *J* = 8.8 Hz, H_6_), 6.85 (d, 3 H, *J* = 8.4 Hz, H_20,24_+NH); 7.33–7.45 (m, 5 H, H_5,13,15,21,23_), 7.51–7.53 (m, 3 H, H_12,14,16_), 7.82 (s, 1H, H_3_), 10.25 (s, 1H, NH). ^13^C NMR (101 MHz, DMSO-*d*_*6*_): δ = 14.8 (C_26_), 24.9 (C_8_), 37.0 (C_7_), 38.8 (C_17_), 63.5 (C_25_), 110.1, 114.6, 117.6, 120.9, 127.5, 130.1, 132.0, 133.0, 136.6, 145.4, 149.8, 154.4, 154.9, 157.1, 165.3 (C_1,3,4,5,6,9−16,18−24_). MS (ESI^+^): *m/z* calculated for C_25_H_25_ClN_6_O_2_S, 509 [M + H]^+^; found 509. Anal. Calcd. (%) for C_25_H_25_ClN_6_O_2_S, C, 58.99; H, 4.95; N, 16.51; S, 6.30. Found: C, 58.84; H, 4.99; N, 16.79; S, 6.36.

##### 2-((5-(2-((3,5-Dibromopyridin-2-yl)amino)ethyl)-4-phenyl-4 H-1,2,4-triazol-3-yl)thio)-N-(4-ethoxyphenyl)acetamide (**30**)

Prepared from 2-bromo-*N*-(4-ethoxyphenyl)acetamide. Yield 0.24 g (59%), brown crystals, m.p. 89–90 °C. IR (KBr) ν_max_ (cm^− 1^): 1573 (C = O), 3215, 3247 (2 NH). ^1^H NMR (400 MHz, DMSO-*d*_*6*_): δ = 1.27 (t, 3 H, *J* = 7.0 Hz, H_26_), 2.79 (t, 2 H, *J* = 6.8 Hz, H_8_), 3.53 (q, 2 H, *J* = 7.2 Hz, *J* = 13.6 Hz, H_7_), 3.94 (q, 2 H, *J* = 7.2 Hz, *J* = 14.0 Hz, H_25_), 4.00 (s, 2 H, H_17_), 6.64 (t, 1H, *J* = 6.0 Hz, NH), 6.84 (d, 2 H, *J* = 8.4 Hz, H_20,24_), 7.34–7.41 (m, 4 H, H_13,15,21,23_), 7.49–7.51 (m, 3 H, H_12,14,16_), 7.87 (s, 2 H, H_3,5_), 10.17 (s, 1H, NH). ^13^C NMR (101 MHz, DMSO-*d*_*6*_): δ = 15.0 (C_26_), 24.7 (C_8_), 30.8 (C_7_), 37.1 (C_17_), 63.6 (C_25_), 104.9, 105.5, 114.9, 121.3, 127.6, 130.3, 132.0, 133.0, 141.5, 146.9, 150.1, 153.6, 154.8, 155.2, 165.6 (C_1,3,4,5,6,9−16,18−24_). MS (ESI^+^): *m/z* calculated for C_25_H_24_Br_2_N_6_O_2_S, 633 [M + H]^+^; found 633. Anal. Calcd. (%) for C_25_H_24_Br_2_N_6_O_2_S, C, 47.48; H, 3.83; N, 13.29; S, 5.07. Found: C, 47.53; H, 3.81; N, 13.27; S, 5.04.

##### N-(4-bromophenyl)-2-((5-(2-((5-chloropyridin-2-yl)amino)ethyl)-4-phenyl-4 H-1,2,4-triazol-3-yl)thio)acetamide (**31**)

Prepared from 2-bromo-*N*-(4-bromophenyl)acetamide. Yield 0.32 g (65%), yellowish brown crystals, m.p. 139–140 °C. IR (KBr) ν_max_ (cm^− 1^): 1594 (C = O), 3184, 3278 (2 NH). ^1^H NMR (400 MHz, DMSO-*d*_*6*_): δ = 2.77 (t, 2 H, *J* = 7.0 Hz, H_8_), 3.44 (q, 2 H, *J* = 6.8 Hz, *J* = 13.2 Hz, H_7_), 4.07 (s, 2 H, H_17_), 6.37 (d, 1H, *J* = 8.8 Hz, H_6_), 6.81 (t, 1H, *J* = 6.0 Hz, NH), 6.96 (t, 1H, *J* = 7.2 Hz, H_13_), 7.27 (t, 1H, *J* = 7.8 Hz, H_15_), 7.38–7.40 (m, 2 H, H_20,24_), 7.44 (m, 2 H, H_21,23_), 7.52–7.55 (m, 4 H, H_5,12,14,16_), 7.82 (s, 1H, H_3_), 10.47 (s, 1H, NH). ^13^C NMR (101 MHz, DMSO-*d*_*6*_): δ = 24.9 (C_8_), 29.7 (C_7_), 37.0 (C_17_), 110.0, 115.4, 117.7, 118.4, 121.3, 127.5, 129.0, 130.2, 131.8, 132.9, 136.6, 138.3, 139.8, 145.4, 149.8, 152.8, 154.5, 157.0, 166.0 (C_1,3,4,5,6,9−16,18−24_). MS (ESI^+^): *m/z* calculated for C_23_H_20_BrClN_6_OS, 545 [M + H]^+^, Br isotopic peak; found 545. Anal. Calcd. (%) for C_23_H_20_BrClN_6_OS, C, 50.79; H, 3.71; N, 15.45; S, 5.89. Found: C, 50.83; H, 3.66; N, 15.41; S, 5.93.

##### N-(4-bromophenyl)-2-((5-(2-((3,5-dibromopyridin-2-yl)amino)ethyl)-4-phenyl-4 H-1,2,4-triazol-3-yl)thio)acetamide (**32**)

Prepared from 2-bromo-*N*-(4-bromophenyl)acetamide. Yield 0.32 g (73%), light grey crystals, m.p. 138–139 °C. IR (KBr) ν_max_ (cm^− 1^): 1585 (C = O), 3238, 3249 (2 NH). ^1^H NMR (400 MHz, DMSO-*d*_*6*_): δ = 2.79 (t, 2 H, *J* = 6.8 Hz, H_8_), 3.52 (q, 2 H, *J* = 9.2 Hz, *J* = 15.6 Hz, H_7_), 4.03 (s, 2 H, H_17_), 6.64 (t, 1H, *J* = 6.0 Hz, NH), 7.36 (d, 2 H, *J* = 4.8 Hz, H_20,24_), 7.45–7.51 (m, 8 H, H_5,12−16,21,23_), 7.88 (s 1H, H_3_), 10.45 (s, 1H, NH). ^13^C NMR (101 MHz, DMSO-*d*_*6*_): δ = 24.7 (C_8_); 30.4 (C_7_), 37.1 (C_17_), 104.9, 105.5, 115.7, 121.6, 127.6, 130.3, 132.1, 133.0, 138.4, 141.5, 146.9, 150.0, 153.6, 154.8, 166.3 (C_1,3,4,5,6,9−16,18−24_). MS (ESI^+^): *m/z* calculated for C_23_H_19_Br_3_N_6_OS, 667 [M + H]^+^; found 667. Anal. Calcd. (%) for C_23_H_19_Br_3_N_6_OS, C, 41.40; H, 2.87; N, 12.60; S, 4.81. Found: C, 41.35; H, 2.82; N, 12.58; S, 4.77.

##### N-(5-chloropyridin-2-yl)-2-((5-(2-((5-chloropyridin-2-yl)amino)ethyl)-4-phenyl-4 H-1,2,4-triazol-3-yl)thio)acetamide (**33**)

Prepared from 2-bromo-*N*-(5-chloropyridin-2-yl)acetamide. Yield 0.39 g (87%), light brown crystals, m.p. 199–200 °C. IR (KBr) ν_max_ (cm^− 1^): 1589 (C = O), 2973, 3238 (2 NH). ^1^H NMR (400 MHz, DMSO-*d*_*6*_): δ = 2.76 (t, 2 H, *J* = 7.0 Hz, H_8_), 3.44 (q, 2 H, *J* = 6.4 Hz, *J* = 13.2 Hz, H_7_), 4.10 (s, 2 H, H_17_), 6.37 (d, 1H, *J* = 8.8 Hz, H_6_), 6.81 (t, 1H, *J* = 6.0 Hz, NH), 7.24 (t, 1H, *J* = 7.8 Hz, H_14_), 7.38–7.40 (m, 2 H, H_22,23_), 7.51–7.53 (m, 3 H, H_5,13,15_), 7.80–7.81 (m, 1H, H_20_), 7.89 (d, 2 H, *J* = 11.6 Hz, H_12_); 8.03 (d, 1H, *J* = 9.2 Hz, H_16_); 8.36 (s, 1H, H_3_), 9.54 (s, 0.3 H, NH), 10.95 (s, 0.7 H, NH). ^13^C NMR (101 MHz, DMSO-*d*_*6*_): δ = 24.9 (C_8_), 36.9 (C_7_), 38.8 (C_17_), 110.0, 114.7, 117.7, 118.4, 121.8, 125.6, 127.5, 128.9, 130.1, 133.0, 136.6, 138.2, 140.3, 145.4, 149.7, 150.5, 154.5, 157.1, 167.0 (C_1,3,4,5,6,9−16,18−24)_. MS (ESI^+^): *m/z* calculated for C_22_H_19_Cl_2_N_7_OS, 500 [M + H]^+^; found 500. Anal. Calcd. (%) for C_22_H_19_Cl_2_N_7_OS, C, 52.81; H, 3.83; N, 19.59; S, 6.41. Found: C, 52.79; H, 3.88; N, 19.66; S, 6.38.

##### N-(5-chloropyridin-2-yl)-2-((5-(2-((3,5-dibromopyridin-2-yl)amino)ethyl)-4-phenyl-4 H-1,2,4-triazol-3-yl)thio)acetamide (**34**)

Prepared from 2-bromo-*N*-(5-chloropyridin-2-yl)acetamide. Yield 0.25 g (61%), grey crystals, m.p. 269–270 °C. IR (KBr) ν_max_ (cm^− 1^): 1583 (C = O), 2927, 3377 (2 NH). ^1^H NMR (400 MHz, DMSO-*d*_*6*_): δ = 2.81 (t, 2 H, *J* = 7.0 Hz, H_8_), 3.55 (q, 2 H, *J* = 6.8 Hz, *J* = 12.8 Hz, H_7_), 4.13 (s, 2 H, H_17_), 6.71 (t, 1H, *J* = 5.8 Hz, NH), 7.43–7.54 (m, 5 H, H_5,13,15,21,23_), 7.89–8.06 (m, 4 H, H_12,14,16,20_), 8.38 (s, 1H, H_3_), 10.97 (s, 1H, NH). ^13^C NMR (101 MHz, DMSO-*d*_*6*_): δ = 24.4 (C_8_), 36.7 (C_17_), 39.2 (C_7_), 104.4, 105.1, 114.5, 125.3, 127.3, 129.8, 132.8, 138.1, 141.1, 146.5, 146.6, 149.4, 150.4, 153.3, 154.3, 166.8 (C_1,3,4,5,6,9−16,18−23_). MS (ESI^+^): *m/z* calculated for C_22_H_18_Br_2_ClN_7_OS, 624 [M + H]^+^; found 624. Anal. Calcd. (%) for C_22_H_18_Br_2_ClN_7_OS, C, 42.36; H, 2.91; N, 15.72; S, 5.14. Found: C, 42.39; H, 2.88; N, 15.66; S, 5.19.

### Biology

#### Anticancer activity

##### Cell culturing

Human lung adenocarcinoma A549, human triple-negative breast cancer MDA-MB-231, and human melanoma IGR39 cell lines were obtained from the American Type Culture Collection (ATCC, Manassas, VA, USA). Human foreskin fibroblasts (HF) CRL-4001 were originally obtained from ATCC and kindly provided by Prof. Helder Santos (University of Helsinki, Helsinki, Finland). Cells were cultured in T25 flasks (Corning Inc., NY, USA) at 37 °C in a humidified atmosphere containing 5% CO_2_. All cells were cultured in Dulbecco’s modified Eagle’s GlutaMAX medium (Gibco (Carlsbad, CA, USA)) supplemented with 1% of 10,000 U/mL penicillin, 10 mg/mL streptomycin (Gibco, Waltham, MA, USA) and 10% fetal bovine serum (Gibco). Cell cultures were used until passage 20.

##### Cell viability assay

Cell viability was evaluated using the 3-(4,5-dimethylthiazol-2-yl)-2,5-diphenyltetrazolium bromide (MTT; Sigma-Aldrich Co., St Louis, MO, USA) reduction assay as described elsewhere elsewhere^63^. Briefly, the cells were seeded into TC-treated flat-bottom 96-well plates (4 × 10^3^ cells/well) and incubated for 24 h. The cells were then treated with 50 µM of the selected compounds and incubated for 72 h. Wells containing medium with 0.5% dimethyl sulphoxide (DMSO; Sigma-Aldrich Co., St. Louis, MO, USA) served as negative controls, and wells without cells served as positive controls. Following incubation, MTT reagent (at a concentration of 0.5 mg/mL) was added to each well and incubated for 3 h. Formazan crystals were dissolved in DMSO, and absorbance was measured at 570 and 630 nm using a multidetection microplate reader Multiscan GO (Thermo Fisher Scientific Oy, Ratastie, Finland).

The EC_50_ values of compounds **24**, **26**, **30**, **31**, **32**, **34**, dasatinib (DST; Sigma-Aldrich Co., St. Louis, MO, USA) and sunitinib (SNT; Sigma-Aldrich Co., St. Louis, MO, USA) were determined using the same MTT assay. Cells were treated with compounds diluted to concentrations ranging from 0.01 µM to 50 µM. The Hill equation was used to calculate the EC_50_ values.

##### Wound Healing Assay

The effect of compounds **24**, **26**, **30**, **31**, **32**, **34**, DST and SNT on cell migration was assessed using the ‘wound healing’ method as described elsewhere^64^. Cancer cells were seeded at a density of 5 × 10^4^ cells/well in 24-well plates and allowed to form confluent monolayers. Scratches were made using a sterile 100 µL pipette tip, and wells were washed with sterile PBS. Fresh medium containing test compounds at 50% and 10% of their respective EC_50_ values was added. Medium with 0.5% DMSO served as the negative control. Images were captured at designated time points (0, 12, 24 h for IGR39 and MDA-MB-231; 0, 24, 48 h for A549) using an Olympus IX73 inverted microscope (Olympus Corporation, Tokyo, Japan).

##### 3D Spheroid Assay

Three-dimensional spheroids were generated using magnetic bioprinting as described elsewhere^27^. Cell cultures were grown to 70% confluence in standard culture conditions. For spheroid generation, these cultures were incubated with magnetic nanoparticles Nanoshuttle (n3D Biosciences, Inc., Houston, TX, USA), for 8 h to facilitate cellular magnetization. Following the incubation period, the cells were detached from the culture vessel using TrypLE (Gibco), collected, and centrifuged. The cells were then resuspended and seeded into ultra-low attachment 96-well plates. To generate heterotypic spheroids that better represent the tumor microenvironment, a co-culture approach was employed, seeding a mixture of 1.5 × 10^3^ cancer cells and 1.5 × 10^3^ human fibroblasts per well. The seeded plate was then placed on a magnetic drive and incubated for two days at 37 °C and 5% CO_2_. Next, medium containing 10 µM of selected compounds (**24**, **26**, **30**, **34**, SNT, DST) was added, and photos were taken using an Olympus IX73 inverted microscope on day 0 and on every other day until day 10. Spheroid size was computed using *AnaSP* spheroid segmentation software^65^.

On the final day of the experiment, cellular viability within the spheroids was assessed using the MTT assay. 20 µL of MTT (5 mg/mL) was added to each well. Following a 10-hour incubation period, the culture medium was aspirated. To ensure complete dissolution of the formazan crystals, 100 µL of DMSO was added to each well, and the plate was incubated overnight. The absorbance was then measured using a multidetector microplate reader, Multiskan GO (Thermo Fisher Scientific Oy, Ratastie, Finland), at 570 nm and 630 nm wavelengths.

##### Antimicrobial activity

The following test microorganisms were used for investigation of antibacterial and antifungal properties of the obtained compounds: bacteria *Escherichia coli B-906*, *Staphylococcus aureus 209-P*, *Mycobacterium luteum B-917* and fungi *Candida tenuis VKM Y-70*, and *Aspergillus niger F-1119*.

The diffusion method^66^ and the serial dilution method^67^ were used for the determination of antimicrobial activity. Vancomycin (antibacterial drug) and nystatin (antifungal drug) were used as the control in the study. The results of antimicrobial evaluation are presented in Tables 2 and 3.

##### Determination of antimicrobial activity of compounds by the diffusion assay

Antibacterial and antifungal action of compounds was evaluated by diffusion in peptone on nutrient medium (meat-extract agar for bacteria, wort agar for fungi). 20 mL of the nutrient medium was added to Petri plates for all test cultures. The microbial loading was 10^9^ cells (spores) / 1 mL. Whatmann filter disks with 0.1 and 0.5% test compound were placed on Petri plates. The duration of incubation was 24 h at 35 °C for bacteria, and 48–72 h at 28–30 °C for fungi. Antimicrobial effect of compounds tested was estimated by measuring the zone diameter (mm) of microorganism growth inhibition (Table 2).

##### Determination of minimal inhibition concentrations (MIC) by the serial dilution assay

The tested compound was dissolved in DMSO in the necessary concentration. A flat-bottomed 96-well tissue culture plates were used for testing. A certain volume of solution of the compound was brought into the nutrient medium (meat-extract agar for bacteria, wort for fungi). The inoculum of bacteria and fungi was inoculated into the nutrient medium. The incubation of bacteria was carried out at 37 °C for bacteria, and at 30 °C for fungi. The duration of incubation was 24–72 h. The results were estimated by the presence or absence of microorganism’s growth in a flat-bottomed 96-well tissue culture plate and are presented as minimal inhibition concentrations MIC (Table 3).

### Molecular docking method

Molecular docking with protein targets for compounds **24**, **26**, **30**, **31**, **32**, and **34** was conducted using the *SchrödingerSuite 2024-1* software package^68^. The following known ten sunitinib-targets were retrieved from the Protein Data Bank (PDB)^69^: SCFR (PDB: 3G0E), ITK (PDB: 3MIY), CDK 2 (PDB: 3TI1), VEGFR2 (PDB: 4AGD), PAK6 (PDB: 4KS8), STK24 (PDB: 4QMZ), PDGFRA (PDB: 6JOK), nonphosphorylated HPK1 (PDB: 6NFY), diphosphorylated HPK1 (PDB: 6NFZ), and HPK1 (PDB: 6NG0). Docking study for the compounds **24**, **26**, **30**, **31**, **32**, and **34** was carried out to the sunitinib-targets mentioned and the structures of target proteins from PDB Bank: EGFR (PDB: 4HJO), VEGFR (PDB: 4AGD, 4ASD, 3EWH), HER2 (PDB: 3RCD), BRAF (PDB: 1UWH, 5VAM, 4G9C), MEK (PDB: 4U7Z), SCR (PDB: 3F3V), CDK5 (PDB: 1UNL). The structures of the kinase inhibitors sunitinib, dasatinib, sorafenib, trametinib, and vemurafenib were obtained from their respective PDB entries, where they were found to bind to the corresponding kinases. The ligands preparation was carried out with the *LigPrep* software^70^. The target proteins were prepared using *Protein Preparation Wizard* software^71^. The ligand-receptor binding site was generated using the Grid Generation of the Glide Maestro module^72^.

### Statistical analysis

All biological experiments were conducted in triplicate unless stated otherwise. Results were expressed as mean ± standard deviation. Data was analyzed using *Microsoft Excel* (Microsoft Corporation, Redmond, WA, USA) and *RStudio* (R Foundation for Statistical Computing, Austria). Statistical significance (*p* < 0.05) was determined using two-way ANOVA and Dunnett’s post hoc.

## Conclusions

4-Phenyl-1,2,4-triazole-3-thiones **9** and **10**, bearing 5-chloro- and 3,5-dibromopyridinyl moieties, were *S*-alkylated with 2-bromo-N-phenylacetamides **17–22** to yield target 1,2,4-triazole-3-thiol derivatives **23–34**, which were evaluated for dual anticancer and antimicrobial activity. Dibrominated 1,2,4-triazole-3-thiol derivatives exhibited the strongest cytotoxicity, with compound **34** emerging as the most potent (EC₅₀ < 5 µM), though still less effective than sunitinib. While the novel compounds did not significantly inhibit cancer cell migration, compounds **24** and **26** reduced the viability of MDA-MB-231 and IGR39 spheroids to a similar extent as both sunitinib and dasatinib, but without altering their size. Several synthesized compounds displayed strong antimicrobial activity, with compounds **24**,** 26**, and **32** showing potent antibacterial effects against *M. luteum* and antifungal activity against *C. tenuis*, comparable to or exceeding standard drugs. Among them, compound **26** exhibited significant antifungal potency. Notably, compounds **24** and **26** also reduced cancer cell viability, with compound **26** emerging as a promising dual-acting anticancer and antimicrobial candidate. Molecular docking suggested possible MEK/BRAF-related interactions for compound **34**. Nevertheless, direct biochemical evaluation against MEK and BRAF kinases is required to validate this hypothesis. Therefore, 1,2,4-triazole-3-thiol derivatives **24**,** 26**, and **34** hold potential as scaffolds for the development of multifunctional therapeutics targeting both infectious and oncological diseases.

## Supplementary Information

Below is the link to the electronic supplementary material.


Supplementary Material 1


## Data Availability

Data is provided within the manuscript or supplementary material files.
