# Supplementary material for: New hybrid pyridine–1,2,4-triazole scaffolds: synthesis, in vitro evaluation of anticancer and antimicrobial activity, and in silico insights
Source: Sci Rep. 2026 May 22;16:27116. doi: 10.1038/s41598-026-53201-3 (PMC13526864; doi:10.1038/s41598-026-53201-3)
Supplement: Supplementary file 1 [file 41598_2026_53201_MOESM1_ESM.docx]

**Supplementary Material**

**New Hybrid Pyridine–1,2,4-Triazole Scaffolds: Synthesis, *In Vitro* Anticancer and Antimicrobial Activity Evaluation, and *In Silico* Insights**

Aida Šermukšnytė^1‡^, Dainius Lukauskas^2‡^, Maryna Stasevych^3^, Viktor Zvarych^4^, Olena Komarovska-Porokhyavets^3^, Kristina Kantminienė^5^*, Ingrida Tumosienė^1^, Vilma Petrikaitė^2,6^*

^1^Department of Organic Chemistry, Kaunas University of Technology, Radvilėnų pl. 19, LT-50254 Kaunas, Lithuania

^2^Department of Drug Targets Histopathology, Institute of Cardiology, Lithuanian University of Health ‎Sciences‎, Lithuania

^3^Department of Technology of Biologically Active Substances, Pharmacy, and Biotechnology, Lviv Polytechnic National University, S. Bandera Str. 12, 79013 Lviv, Ukraine

^4^Department of Automated Control Systems, Lviv Polytechnic National University, S. Bandera Str. 12, 79013 Lviv, Ukraine

^5^Department of Physical and Inorganic Chemistry, Kaunas University of Technology, Radvilėnų pl. 19, LT-50254

^6^Institute of Biotechnology, Life Sciences Center, Vilnius University, Saulėtekio 7, LT-10257 Vilnius, Lithuania

Corresponding authors

*E-mail: [kristina.kantminiene@ktu.lt](mailto:vytautas.mickevicius@ktu.lt), vilma.petrikaite@lsmu.lt

^‡^A.Š. and D.L. contributed equally to this work.

**Figure S1.** ^1^H NMR (400 MHz, DMSO-d_6_) spectrum of **3**.

**Figure S2.** ^13^C NMR (101 MHz, DMSO-d_6_) spectrum of **3**.

**Figure S3.** ^1^H NMR (400 MHz, DMSO-d_6_) spectrum of  **4.**

**Figure S4.** ^13^C NMR (101 MHz, DMSO-d_6_) spectrum of **4.**

**Figure S5.** ^1^H NMR (400 MHz, DMSO-d_6_) spectrum of **5**.

**Figure S6.** ^13^C NMR (101 MHz, DMSO-d_6_) spectrum of **5**.

**Figure S7**. ^1^H NMR (400 MHz, DMSO-d_6_) spectrum of **6**.

**Figure S8.** ^13^C NMR (101 MHz, DMSO-d_6_) spectrum of **6.**

**Figure S9.** ^1^H NMR (400 MHz, DMSO-d_6_) spectrum of **7**.

**Figure S10.** ^13^C NMR (101 MHz, DMSO-d_6_) spectrum of **7.**

**Figure S11.** MS spectrum of **7.**

**Figure S12.** ^1^H NMR (400 MHz, DMSO-d_6_) spectrum of **8.**

**Figure S13.** ^13^C NMR (101 MHz, DMSO-d_6_) spectrum of **8.**

**Figure S14.** MS spectrum of **8.**

**Figure S15.** ^1^H NMR (400 MHz, DMSO-d_6_) spectrum of **9.**

**Figure S16.** ^13^C NMR (101 MHz, DMSO-d_6_) spectrum of **9.**

**Figure S17.** MS spectrum of **9.**

**Figure S18.** ^1^H NMR (400 MHz, DMSO-d_6_) spectrum of **10.**

**Figure S19.** ^13^C NMR (101 MHz, DMSO-d_6_) spectrum of **10.**

**Figure S20.** MS spectrum of **10.**

**Figure S21**. ^1^H NMR (400 MHz, DMSO-d_6_) spectrum of **23.**

**Figure S22**. ^13^C NMR (101 MHz, DMSO-d_6_) spectrum of **23.**

**Figure S23.** MS spectrum of **23.**

**Figure S24.** ^1^H NMR (400 MHz, DMSO-d_6_) spectrum of **24.**

**Figure S25.** ^13^C NMR (101 MHz, DMSO-*d_6_*) spectrum of **24.**

**Figure S26.** MS spectrum of **24.**

**Figure S27.** ^1^H NMR (400 MHz, DMSO-d_6_) spectrum of **25.**

**Figure S28.** ^13^C NMR (101 MHz, DMSO-d_6_) spectrum of **25.**

**Figure S29.** MS spectrum of **25.**

**Figure S30.** ^1^H NMR (400 MHz, DMSO-d_6_) spectrum of **26.**

**Figure S31.** ^13^C NMR (101 MHz, DMSO-d_6_) spectrum of **26.**

**Figure S32.** MS spectrum of **26.**

**Figure S33.** ^1^H NMR (400 MHz, DMSO-d_6_) spectrum of **27.**

**Figure S34.** ^13^C NMR (101 MHz, DMSO-d_6_) spectrum of **27.**

**Figure S35.** MS spectrum of **27.**

**Figure S36.** ^1^H NMR (400 MHz, DMSO-*d_6_*) spectrum of **28**

**Figure S37.** ^13^C NMR (101 MHz, DMSO-d_6_) spectrum of **28.**

**Figure S38.** MS spectrum of **28.**

**Figure S39.** ^1^H NMR (400 MHz, DMSO-d_6_) spectrum of **29.**

**Figure S40.** ^13^C NMR (101 MHz, DMSO-d_6_) spectrum of **29.**

**Figure S41.** MS spectrum of **29.**

**Figure S42.** ^1^H NMR (400 MHz, DMSO-d_6_) spectrum of **30.**

**Figure S43.** ^13^C NMR (101 MHz, DMSO-d_6_) spectrum of **30.**

**Figure S44.** MS spectrum of **30.**

**Figure S45.** ^1^H NMR (400 MHz, DMSO-d_6_) spectrum of **31.**

**Figure S46.** ^13^C NMR (101 MHz, DMSO-d_6_) spectrum of **31**

**Figure S47.** MS spectrum of **31.**

**Figure S48.** ^1^H NMR (400 MHz, DMSO-d_6_) spectrum of **32.**

**Figure S49.** ^13^C NMR (101 MHz, DMSO-d_6_) spectrum of **32.**

**Figure S50.** MS spectrum of **32.**

**Figure S51.** ^1^H NMR (400 MHz, DMSO-d_6_) spectrum of **33.**

**Figure S52.** ^13^C NMR (101 MHz, DMSO-d_6_) spectrum of **33.**

**Figure S53.** MS spectrum of **33.**

**Figure S54.** ^1^H NMR (400 MHz, DMSO-d_6_) spectrum of **34.**

**Figure S55.** ^13^C NMR (101 MHz, DMSO-d_6_) spectrum of **34.**

**Figure S56.** MS spectrum of **34.**

**Figure S57**. Influence of compounds **24**, **26**, **30**, **34**, dasatinib (DST), and sunitinib (SNT) on human lung adenocarcinoma A549 (A), human triple negative breast cancer MDA-MB-231 (B), human melanoma IGR39 (C) migration at 10% of their respective EC_50_ concentrations, n = 3. Statistical significance is denoted with an asterisk (*) for values p < 0.05.

**Figure S58**. Representative images of A549 spheroids every two days of the experiment until day 10. DST – dasatinib.

**Figure S59**. Representative images of MDA-MB-231 spheroids every two days of the experiment until day 10. DST – dasatinib.

**Figure S60**. Representative images of IGR39 spheroids every two days of the experiment until day 10. DST – dasatinib.

**Table S1.** Docking score values of **24**, **26**, **30**, **31**, **32**, and **34** ligands and sunitinib interaction with sunitinib-target protein kinases (part 1)

| Ligand | Protein kinases sites | | | | | | | | | |
| --- | --- | --- | --- | --- | --- | --- | --- | --- | --- | --- |
|  | PDB: 3G0E | | PDB: 3MIY | | PDB: 3TI1 | | PDB: 4AGD | | PDB: 4KS8 | |
|  | Docking Score  kcal/mol | Glide Emodel kcal/mol | Docking Score  kcal/mol | Glide Emodel kcal/mol | Docking Score  kcal/mol | Glide Emodel kcal/mol | Docking Score  kcal/mol | Glide Emodel kcal/mol | Docking Score  kcal/mol | Glide Emodel kcal/mol |
| **24** | -7.321 | -82.914 | -7.148 | -69.526 | -6.770 | -72.006 | -6.057 | -70.810 | -6.111 | -68.180 |
| **26** | -7.978 | -87.978 | -6.327 | -65.385 | -5.522 | -73.128 | -7.000 | -75.575 | -5.767 | -70.278 |
| **30** | -7.659 | -85.699 | -6.564 | -71.248 | -5.431 | -64.830 | -6.466 | -72.575 | -7.046 | -76.188 |
| **31** | -5.835 | -69.105 | -5.813 | -64.796 | -5.288 | -70.706 | -7.504 | -83.147 | -5.340 | -65.682 |
| **32** | -7.978 | -87.978 | -6.334 | -71.248 | -5.501 | 73.246 | -6.458 | -69.487 | -6.378 | -73.188 |
| **34** | -7.677 | -89.997 | -7.375 | -72.414 | -5.859 | -79.314 | -6.033 | -61.539 | -6.261 | -76.580 |
| Sunitinib | **-11.612** | **-106.955** | **-11.892** | **-112.223** | **-10.309** | **-93.624** | **-10.655** | **-91.699** | **-10.596** | **-99.478** |

**Table S2**. Docking score values of **24**, **26**, **30**, **31**, **32**, and **34** ligands and sunitinib interaction with sunitinib-target protein kinases (part 2)

| Ligand | Protein kinases sites | | | | | | | | | |
| --- | --- | --- | --- | --- | --- | --- | --- | --- | --- | --- |
|  | PDB: 4QMZ | | PDB: 6JOK | | PDB: 6NFY | | PDB: 6NFZ | | PDB: 6NG0 | |
|  | Docking Score  kcal/mol | Glide Emodel kcal/mol | Docking Score  kcal/mol | Glide Emodel kcal/mol | Docking Score  kcal/mol | Glide Emodel kcal/mol | Docking Score  kcal/mol | Glide Emodel kcal/mol | Docking Score  kcal/mol | Glide Emodel kcal/mol |
| **24** | -6.515 | -76.619 | -6.854 | -73.813 | -6.856 | -75.694 | -5.768 | -72.685 | -6.002 | -69.576 |
| **26** | -6.016 | -65.180 | -6.141 | -71.753 | -5.904 | -67.031 | -5.837 | -70.867 | -5.334 | -59.632 |
| **30** | -6.113 | -65.008 | -6.940 | -82.874 | --5.285 | -69.006 | -6.483 | -76.631 | -6.342 | -68.175 |
| **31** | -5.793 | -63.217 | -6.650 | -71.948 | -6.680 | -79.461 | -4.860 | -68.519 | -5.852 | -74.193 |
| **32** | -6.561 | -74.240 | -7.098 | -80.643 | -5.774 | -72.494 | -6.343 | -78.160 | -6.819 | -80.233 |
| **34** | -6.440 | -72.218 | -7.185 | -83.309 | -5.581 | -71.079 | -6.569 | -75.665 | -4.487 | -59.997 |
| Sunitinib | **-11.035** | **-110.072** | **-9.706** | **-87.952** | **-10.760** | **-104.769** | **-9.414** | **-92.743** | **-10.168** | **-103.948** |
